# One Health approach for elimination of human anthrax in a tribal district of Odisha: Study protocol

**DOI:** 10.1371/journal.pone.0251041

**Published:** 2021-05-27

**Authors:** Debdutta Bhattacharya, Jaya Singh Kshatri, Hari Ram Choudhary, Debaprasad Parai, Jyoti Shandilya, Asit Mansingh, Matrujyoti Pattnaik, Kaushik Mishra, Shakti Prakash Padhi, Arun Padhi, Sanghamitra Pati

**Affiliations:** 1 ICMR – Regional Medical Research Centre, Bhubaneswar, Odisha, India; 2 Saheed Laxman Nayak Medical College & Hospital, Koraput, Odisha, India; 3 Department of Public Health, Government of Odisha, Bhubaneswar, Odisha, India; 4 Department of Public Health, Koraput, Odisha, India; IAVI, UNITED STATES

## Abstract

**Background:**

Anthrax is a major but neglected zoonotic disease of public health concern in India with Odisha contributing a major share to the disease burden. *Bacillus anthracis* spores can be found naturally in soil and commonly affect both animals and humans around the world. Domestic and wild animals such as cattle, sheep, goats, and deer can become infected when they inhale or ingest spores from contaminated soil, plants, or water. Anthrax can be fatal if patients are not treated promptly with antibiotics. This protocol aims to describe the implementation and evaluation of the ‘One Health’ intervention model based on the principles of Theory of Change (ToC) to eliminate human anthrax from a tribal district in Odisha, India.

**Methods:**

This study would test the effectiveness of a complex public health intervention package developed using the ToC framework for the elimination of human anthrax in Koraput district by a comparative analysis of baseline and end-line data. We plan to enroll 2640 adults across 14 geographically divided blocks in Koraput district of Odisha for baseline and end-line surveys. After baseline, we would provide capacity building training to stakeholders from the department of health, veterinary, forest, academic and allied health institutions followed by workshops on sensitization and awareness through IEC (Information Education Communication)/BCC (Behavior Change Communication) activities in the community. We would establish a state-level laboratory facility as a robust system for timely diagnosis and management of human anthrax cases. Surveillance network will be strengthened to track the cases in early stage and risk zoning will be done for focused surveillance in endemic areas. Advocacy with district level administration will be done for maximizing the coverage of livestock vaccination in the entire district. Interdepartmental coordination would be established for the effective implementation of the intervention package.

**Conclusion:**

This would be a first study applying One Health concept for the elimination of human anthrax in India. The findings from this study will offer important insights for policy-making and further replication in other endemic regions of the state and country.

**Trial registration:**

The authors confirm that all ongoing and related trials for this intervention are prospectively registered with the Clinical Trials Registry of India [CTRI/2020/05/025325] on 22 May 2020.

## Background

Zoonoses are recognized as a major concern in developing nations, in need of a resolution [1]. It is commonly agreed that the detection, management, and elimination of zoonotic diseases requires joint efforts from the animal and human health sectors [2, 3]. Joint monitoring systems such as “One Health” strategy are the most efficient and reliable means of addressing zoonotic diseases such as anthrax in low-income countries [4]. Most of these countries are lacking an integrated control system to address gaps across human health, animal health, food and environment sectors with limited collaborative works. As a result, elimination of a zoonotic diseases remains an unsuccessful goal due to the lack of specific investigation protocol, insufficient knowledge and weak coordination among the responsible stakeholders [5]. The One Health approach mainly relies on vigorous examination of evidence for proof of concept which can be achieved through in-person meetings, circulation of meeting summaries and establishment of standard operating procedures. This concept is already found to have significance when implemented to tackle the newly emerging diseases like Middle East respiratory syndrome, avian flu (H5N1), swine flu (H1N1), Ebola and Zika virus [6, 7]. Management and effective control of anthrax through One Health concept is described as a highly demanding approach although it needs a robust implementation globally [8].

Zoonotic diseases have a significant impact on India’s public health, animal economies, and wildlife. Among these diseases, anthrax contributes to most cases [9]. Anthrax is a widespread zoonotic disease that mainly affects herbivorous animals, triggered by rod-shaped, gram-positive bacteria known as *Bacillus anthracis*. [10, 11] Anthrax most often arises in warm-blooded wild or domesticated livestock such as sheep, cattle, and goats but can cause disease in humans, inducing three forms of the lung (pulmonary), digestive (intestinal), or skin (cutaneous) infections. Ultimately all forms of anthrax may spread across the body and cause death if not properly treated with antibiotics at the right time. Anthrax is known to be a hot-season disease and outbreaks continue to occur in hot-dry climates accompanied by occasional brief showers [12].

India is an endemic country for animal anthrax owing to a large and unsafe population of livestock leading to the outbreaks of human cases in certain parts [13, 14]. In many states of India, anthrax is enzootic including Andhra Pradesh, Jammu and Kashmir, Tamil Nadu, Odisha, and Karnataka [15]. The real burden of anthrax in India is not understood precisely since a significant number of cases go unreported and only a portion of human cases seek medical care [16].

Over the last 15 years, 14 districts out of 30 revenue districts in Odisha have experienced outbreaks of human anthrax with 1208 cases and 436 deaths [17, 18]. Among those affected districts, Koraput has more than 300 human anthrax cases including 10 deaths over the past 6 years. Almost all the cases came from a group of indigenous tribes involved in handling and consuming animal carcasses. Therefore, this implementation research project is planned in the district of Koraput using the "One Health" approach to demonstrate the feasibility for the elimination of human anthrax cases in three years.

### Objectives

The primary objective of this study is to develop and implement a package of coordinated interventions using the “One Health” approach to demonstrate a replicable strategy for the elimination of animal-to-human disease transmission of anthrax in an endemic district of Odisha.

Secondary objectives include assessment of disease burdens, patterns, knowledge, prevalence, and practices among the identified stakeholders from the departments of health, animal husbandry, environment/forest, and the community; and to evaluate the effectiveness of the interventions among the study population on anthrax burden in the study district along with cost-benefit analysis of interventions.

## Methods

### Study design

This is an implementation-effectiveness hybrid design implementation research with a single-arm interventional study built-in to test the effectiveness of structured public health interventions. This includes a phase of formative research including mapping of the project area, development of intervention packages using “Theory of Change” principles and preparing communities & stakeholders for the planned interventions. Phase II entails the implementation and evaluation of the intervention package, using an iterative process of community trials. Qualitative methods would be used to evaluate the feasibility and acceptability of the interventions among the implementers and target population.

### Study setting

**Fig 1:**
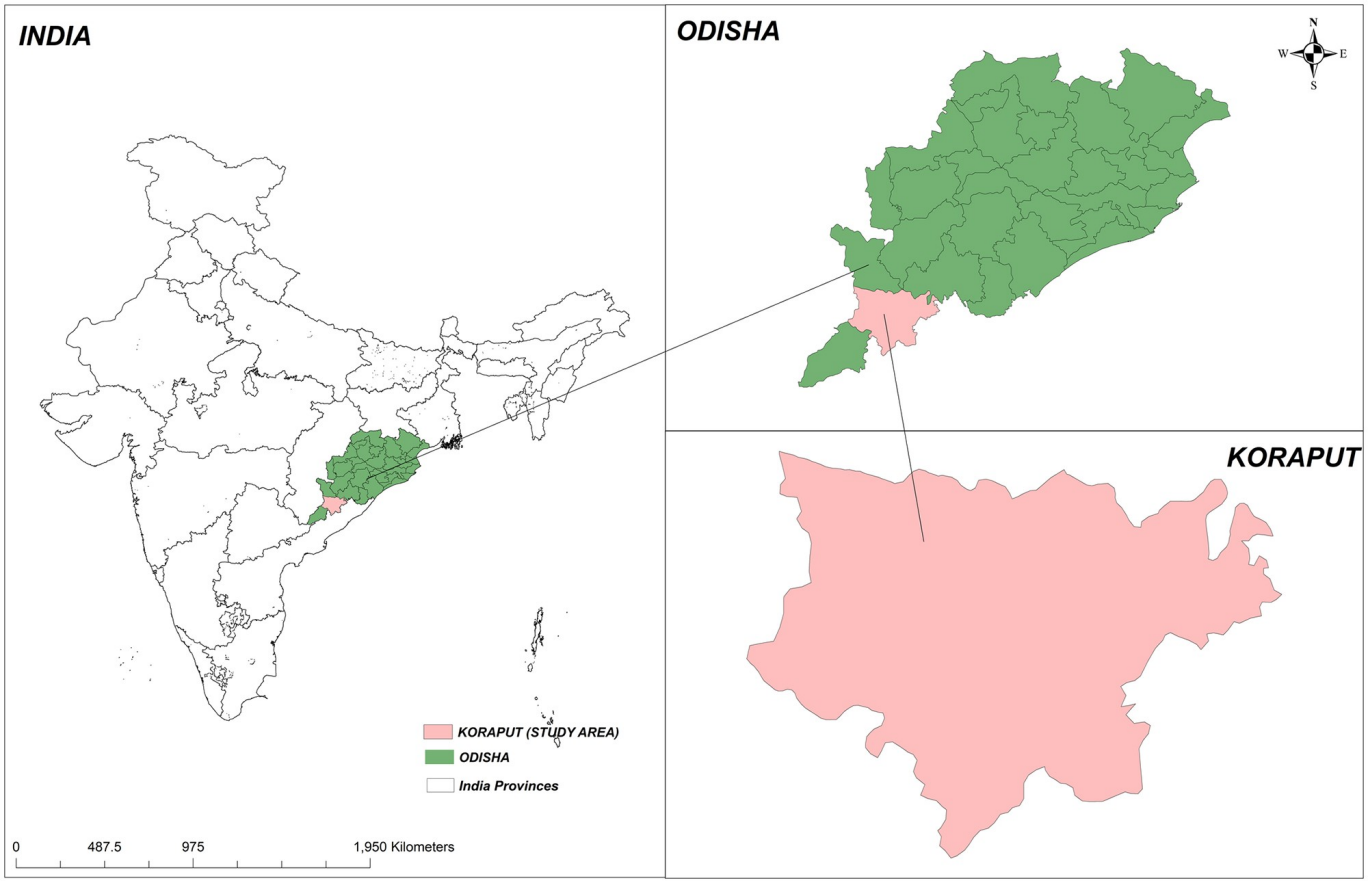
Study setting map.

This study will be carried out in the Koraput District (which has a population of 1.37 million residents across 2028 villages) reporting the highest number of human anthrax cases in Odisha state [18, 19]. Within the district, it will be executed in all geographically divided 14 administrative blocks, which comprise 240 village administrative bodies or ‘gram panchayats’; this includes many tribal villages based in hilly areas [Fig 1]. Eight villages from each block were selected randomly for baseline and end-line assessment surveys of the community trials.

### Study participants

Participants will be enrolled by following the inclusion and exclusion criteria given below:–

Inclusion criteria: Participants will be selected based on the eligibility criteria: a) Should be an adult (age above 18 years) and a permanent resident of Koraput district, b) willing to participate in the study.
Exclusion criteria: a) Cognitive impairment, b) having onset symptoms or infected with any of the communicable disease which can be spread through nasal or mouth droplets.

The study participants for the baseline and end-line study will be sampled from the stakeholders identified in the district. A comprehensive stakeholder mapping and analysis will be carried out based on the broad categories outlined in [Table 1].

**Table 1:** Identified groups of stakeholders to be included in the study.

Health Care Sector	Animal Care Sector	Community
**Clinical service providers**	Veterinary doctors	Service utilizer clients
**Program managers**	Livestock inspectors	Local governance members
**Health workers**	Forest guards	Non-governmental organizations and Self-help groups
**Accredited social health activists (ASHAs)**		Cattle owners/grazers
		Hunter/gatherers
		Village residents

Additionally, participants for the qualitative study will be selected purposively from the known hotspots of outbreaks and tribal communities with practices that make them vulnerable for anthrax infection, acquisition, and propagation.

### Sample size determination

The sample size required for the quantitative component of the baseline (and end-line) study was estimated to be 2608. For uniformity of sampling, this was rounded off to 2640 in total. This was calculated by the following formula:

$$\text{The}\;\text{minimum}\;\text{sample}\;\text{size}\;\text{required}=\frac{\left[(1.96{)}^{z}*P*(1-P)\right]}{d^{2}}*(\text{Design}\;\text{effect})$$

Where the level of confidence assumed at 95%, corresponding to Z = 1.96; P = Prevalence of high-risk exposure to anthrax (Assumed at 5%); d = Relative precision at 20% of P; Design effect = 1.3; Non-Response rate = 10%. For the qualitative component, data collection would be continued until saturation is reached based on the principle of maximum divergence [20].

### Ethics approval and consent to participate

The institutional review board of the ICMR—Regional Medical Research Centre, Bhubaneswar approved this study. The Indian Council of Medical Research has reviewed and approved the study design and protocols. All participants will receive written information on study aims, participation requirements, and the right to refuse. Furthermore, they will complete the written informed consent process before participation.

### Theoretical framework

We would use the Theory of Change (ToC) principle to build and test the complex intervention model for the said objectives. Theory of Change articulates the change process and describes how interventions can bring about long-term outcomes through a logical sequence of intermediate outcomes [21]. Thus, we have adopted this concept to empower and enable the various stakeholders to build interventions to bring about behaviour change among community members and systems. ToC frameworks emphasize on how a complex intervention interacts with the wider system rather than being considered only in the context of one sector [18].

### Overview of the intervention package

The interventions will be developed through a consultative process, requiring stakeholders to reflect on how their programs can bring change. The following broad interventions will be developed and tested:

- Strengthening the health care system and surveillance system for early reporting of suspected cases of human and animal anthrax by developing and implementing the “One-Health” bio-surveillance system.
- Establishment of an anthrax diagnosis facility in the state to facilitate early diagnosis and reporting of anthrax cases based on standardized case definitions.
- Operationalization of standard operating procedures and outbreak response protocols for all stakeholder departments in a consultative manner.
- Provision of postexposure prophylaxis of suspected contacts by the health department at the health facility and community.
- Livestock vaccination program by the state on a large scale and strategy of “ring vaccination” during outbreaks followed by a cost-benefit analysis of the same.
- Placing a framework for categorization of geographical “Risk-Zones” based on GIS mapping of cases for prioritization of public health interventions.
- Capacity building of stakeholders for early detection and appropriate action at district, sub-district, block, and village level, including clinical case management and referral.
- Targeted and generalized IEC/BCC activities to be implemented round-the-year by the state departments in a coordinated manner.

### Study procedures

We will use a team approach, with external and domain expert representation for the development and effective implementation of our intervention model. Please see [Table 2] for the schedule of enrollment, intervention, and assessment. The details of the teams and their compositions, responsibilities, and activities are provided in [S1 Appendix]. The interventions would be implemented in the entire district in partnership with various departments of the state (Health, Animal resources, Forest, and Environment & local governance) in the following manner:

- Developing a “Risk zoning” protocol based on surveillance data available on the previous number of cases (both human and animal) from different units in the district. Geographic Information systems mapping will be done on a real-time basis to categorize the geographical limits of the district into a RED (Action), YELLOW (Alert), and GREEN (Monitor) zones during and following an outbreak. Capacity building, surveillance, and IEC/BCC activities shall depend on the prioritization zone of the location.
- Advocacy for a strong free-of-cost routine and outbreak (ring) vaccination program in the district using findings of the cost-benefit analysis, desk review, and baseline study.
- Development of departmental and interdepartmental Standard Operating Procedures for both exclusive and inter-operable activities and conduct of multi-departmental coordination meetings.
- Development of training and capacity building materials for each stakeholder group, finalization of training calendar, and micro plan followed by conduction of Training for capacity building among Primary Stakeholders from different departments (Health, Veterinary, Forest & Environment, local governance bodies). Training on case definition, early diagnosis, reporting cases, sample collection will be done at the district level.
- Development of IEC material for each group of stakeholders and assisting the state in carrying out IEC and BCC activities in the community. BCC strategy will be developed using Social Cognitive Theory, which depicts that behavior change in humans can be explained by a triadic interaction of behavior, personal and environmental factors [22].
- Strengthening of surveillance for anthrax by developing surveillance protocols by adaptation of WHO protocols, testing, and implementing the surveillance system, and facilitating a regional diagnostics service for anthrax as per the case definitions and diagnostics protocol provided in [S2 Appendix].

**Table 2:** Schedule of enrollment, intervention, and assessments.

	Year 1		Year 2		Year 3	
TIMEPOINT**	Q. 1 &2	Q. 3 & 4	Q. 1 &2	Q. 3 & 4	Q. 1 &2	Q. 3 & 4
ENROLLMENT:						
• *Eligibility Screen*	X					
• *Informed Consent*	X					
• *Baseline Survey*	X					
INTERVENTIONS:						
• *Interventions Implementations*		X	X	X		
ASSESSMENTS:						
• *End line Survey [baseline variables*: *see* Table 3*]*					X	
• *Qualitative assessment of the effectiveness of public health interventions*						X

### Multilevel multi-departmental coordination

**Fig 2:**
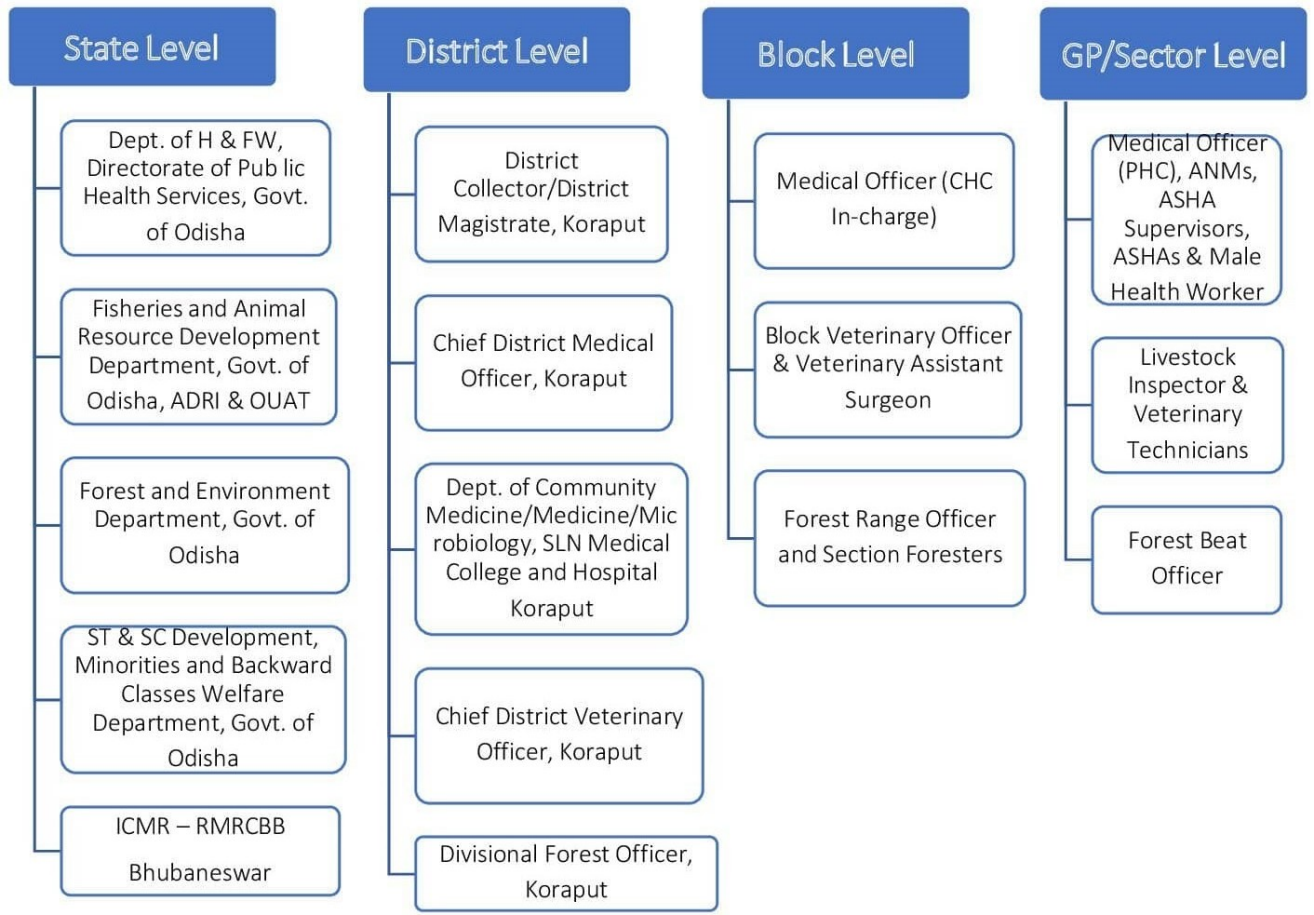
Stakeholders for multilevel multi-departmental coordination.

Multi-departmental coordination is an essential component for effective implementation of our intervention model and therefore we have developed the multi-departmental coordination teams at multi-levels which will ensure the departmental and multi-departmental coordination of planned activities. The figure given below may give you an overall picture to understand the actual coordination at different levels among different departments [Fig 2].

### Study timeline

#### Participant selection

A list of total Gram Panchayats and total number of villages will be made for all the fourteen blocks. From each block two Gram Panchayats, and four villages from each Gram Panchayat will be selected based on simple random sampling using random number generation method. In this way, 2640 individuals from 112 villages will be selected for quantitative data collection during baseline and end line. Systematic random sampling will be used to select the households for the survey in the selected villages. Beginning of the survey will be from the middle point of the village and only one individual will be enrolled as participant from each house. The randomization process from district to household level at each step will ensure that the sample size selected for our study will be representative for the larger population of Koraput.

Quantitative data will be collected regarding the knowledge of the anthrax disease, attitudes towards curbing it, and practices among villagers that led to the rise of cases as given in Table 3. Data will be collected using electronic data capture tools. Enrolment of participant has started from 16^th^ June 2020 and will be finished by the month of October 2020. End-line study will take place as per the schedule mentioned in the study timeline [see Table 2]

**Table 3:** Content of the baseline survey.

Topics and Constructs	Description
Socio-economic and demographic characteristics	Gender, age, residency, education, religion, caste, household members, occupation, family income etc.
Information on domestic animals	Existence of livestock, number of livestock, types, the purpose of keeping livestock, place of keeping livestock, involvement with livestock, the place for grazing, frequency of taking for grazing, the experience of animal handling, dispose of solid and animal waste products
Vaccination	Awareness about animal vaccination, the practice of vaccination, type of animals vaccinated, attitude towards animal vaccination, frequency of vaccination, reasons for not getting animals vaccinated, place of vaccination, the cost for vaccination
Practices of animal handling	Practices of dead animals’ burial, symptoms observations during deaths, information on recent animal death, de skinning practices with dead animals, use of protective equipment for skinning, govt. financial support for burial and death compensation
Food habits	Consumption of meat, type of meat they consume, sources of getting meat, ways of consuming, frequency, preservation of meat, consumption of animal blood
Knowledge and awareness about anthrax	General awareness, source of information, Local names, transmission, behavior/practices with suspected and dead animals due to anthrax, reporting structure, ways of treatment, information of cases in recent past, activities are done for awareness, knowledge about human anthrax, symptoms in humans, experience with human anthrax

Focus group discussions (FGDs) from 4 blocks (2 endemics and 2 nonendemic) blocks of the district will be carried out to provide insight into the process of intervention delivery, how it affects behavior and practices changes, and how this model is perceived and valued. Key person interviews and in-depth interviews will be carried out to assess the feasibility and identify the bottlenecks in the implementation processes from the systems side as well.

### Data analysis

Effectiveness of our complex public health intervention package will be analyzed as a whole One Health strategy instead of its separate components for elimination of human anthrax cases in endemic regions. Furthermore, the impact of each intervention will be tracked through the logic model developed by us with different levels such as inputs, activities, outputs, indicators, outcomes & impact [Table 4 in S3 Appendix]. Our quantitative and qualitative surveys during baseline and end line will be analyzed to observe the effectiveness of implementation of our intervention package and to observe the improvement in knowledge, behavior, and practices with regards to the anthrax disease among communities residing in the Koraput district. Annual data of animal vaccination, animal and human anthrax cases and deaths will also be an analysis to see the progress and track the effective implementation of our intervention package. The data of human anthrax cases and deaths will be taken from IDSP (Integrated Diseases Surveillance Program) Odisha and similarly the data of animal vaccination coverage, animal anthrax cases and deaths will be taken from Veterinary department in the district. At the end of the 3 years, we will do a comparative analysis of vaccination coverage, animal and human anthrax cases and deaths in the district to find the effectiveness of our interventions. Descriptive statistics will be used for the analysis of quantitative data though SPSS (Statistical Package for Social Sciences) or STATA. Qualitative data will be analyzed through thematic analysis, and network diagrams. The primary comparison of baseline and end-line data will be made by applying tests of statistical significance where applicable, with a level of significance established at p = 0.05. Moreover, bottleneck analysis will be done using appropriate models for the surveillance system in place and finding of this study will be published in open access journal upon completion of study [23].

## Discussion

The study is for three years based on implementation models including surveys (baseline and end line), capacity building, and sensitization of various stakeholders from the department of health, veterinary, and forest systems at various levels in a tribal district of Odisha, India. The prevalence and outbreaks of anthrax are interlinked with the animal-environment-human context, which signifies the need for collaborative, transdisciplinary, and multi-sectoral approaches for the prevention and control of anthrax [24]. The intervention builds upon a preliminary study to strengthen the health care and surveillance system for early reporting of suspected cases of human and animal anthrax by developing and implementing the “One-Health” strategy. We aim to eliminate incidences of human anthrax cases to zero by the end of three years using a multi-sectoral team-based approach. We also propose for sustainable on-ground investigation and surveillance after this intervention-implementation in collaboration with health, veterinary, forest and other allied departments in the district and state. Effectiveness of the capacity building training for enhancing knowledge will be assessed by structured pre- and post-assessments. Findings from this study would provide recommendations for policymaking and future scale-up planning to the elimination of zoonotic diseases using ‘One Health’ strategies. Moreover, the study protocol may further be replicated in other endemic regions of India as well as other countries.

## Supporting information

S1 Appendix Roles and responsibilities of project teams. (PDF)

S2 Appendix Surveillance protocol. (PDF)

S3 Appendix Project proposal. (PDF)

S4 Appendix State ethics committee approval document. (PDF)

S5 Appendix Institutional ethics committee approval document. (PDF)

S6 Appendix WHO trail registration document. (PDF)

S7 Appendix SPIRIT checklist. (DOC)

S8 Appendix Baseline survey questionnaire. (PDF)

## Abbreviations

ANM: Auxiliary Nurse Midwife
ASHA: Accredited Social Health Activist
BCC: Behaviour Change Communication
CBT: Capacity Building Training
CDMO: Chief District Medical Officer
CDVO: Chief District Veterinary Officers
CHC: Community Health Centre
CTRI: Clinical Trial Registry- India
FHW: Female Health Worker
GIS: Geographic Information System
IDSP: Integrated Disease Surveillance Programme
IEC: Information Education Communication
MDC: Multi Departmental Coordination
MHW: Male Health Worker
MO: Medical Officer
MPH: Master of Public Health
NCDC: National Centre for Disease Control
NIVEDI: National Institute Of Veterinary Epidemiology And Disease Informatics
PCR: Polymerase Chain Reaction
PHC: Public Health Centre
RMRC: Regional Medical Research Centre
SC: Sub-Centre
ToC: Theory of Change
