# Supplementary material for: One Health approach for elimination of human anthrax in a tribal district of Odisha: Study protocol
Source: PLoS One. 2021 May 27;16(5):e0251041. doi: 10.1371/journal.pone.0251041 (PMC8158997; doi:10.1371/journal.pone.0251041)
Supplement: S1 Appendix — (PDF) [file pone.0251041.s001.pdf]

S1 Appendix. Roles and Responsibilities of Project Teams

Team Name	Team members	Activities
Project Management Team	PI, Co-PIs, External Expert Advisor	Formation of study teams.<br>Carrying out the IEC/BCC campaign.<br>Finalization of training calendar.<br>Calendar of MDC meetings.<br>Risk Zoning.
Monitoring and Evaluation Team	Scientist-C (Public Health), Research Assistant, External Expert (Veterinary), External Expert (Health System)	Conducting quality monitoring of other team's activities.<br>Development of evaluation reports.
Technical Advisory Team	PI, Co-PIs, Domain Experts (1 Veterinarian, 1 Physician, 1 Public health specialist, 1 Microbiologist), Scientist-B (Public Health) Scientist-B (Non-medical)	Development of surveillance protocols.<br>Development of SOPs.<br>Development of IEC materials.<br>Development of training & capacity building materials.
Capacity Building and Training Team	Co-PIs, Research Assistant, Faculty members from partner agencies, Scientist-B (Public Health) Scientist-B (Non-medical)	Development of training & capacity building materials.<br>Conduct of training.<br>Finalization of training calendar.
Multi-Departmental Co-Ordination Team	Research Assistant, Scientist-B (Public Health) Scientist-B (Non-medical)	Development of SOPs.<br>Calendar of MDC meetings.<br>Conduct of MDC meetings.
Integrated Surveillance Team	Co-PI, Research Assistant, Field Investigators, Data Entry Operators, External experts from Bio-surveillance and Public Health surveillance.	Development of surveillance protocols.<br>Testing and implementing a surveillance system.
