# Supplementary material for: One Health approach for elimination of human anthrax in a tribal district of Odisha: Study protocol
Source: PLoS One. 2021 May 27;16(5):e0251041. doi: 10.1371/journal.pone.0251041 (PMC8158997; doi:10.1371/journal.pone.0251041)
Supplement: S2 Appendix — (PDF) [file pone.0251041.s002.pdf]

S2 Appendix. Surveillance Protocol

<u>B. anthracis *case definitions:*</u>

Suspect: A case that meets the clinical criteria, BUT with no presumptive or confirmatory laboratory evidence, or epidemiologic evidence relating it to anthrax

Probable: A case that meets the clinical criteria AND has presumptive laboratory test results

Confirmed: A case that meets the clinical criteria AND has confirmatory laboratory test results

<u>The testing algorithm</u>:

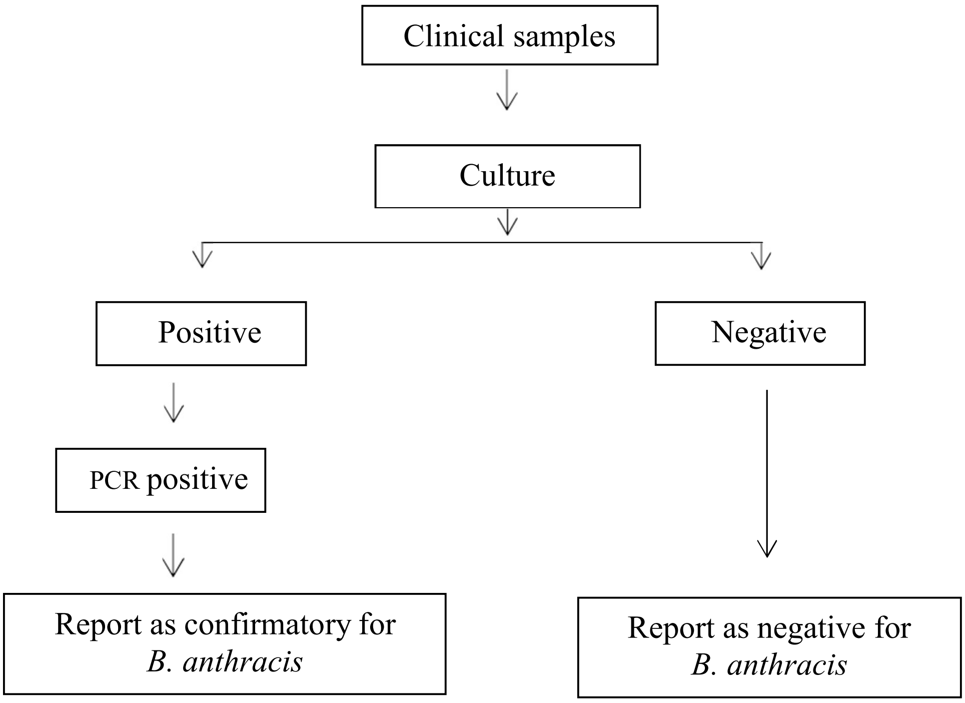

<u>Sample collection</u>:

Samples should be collected using sterile swabs in duplicate (for culture & PCR) from

a) Vesicular Lesions
b) Eschers
c) Ulcers

The swabs should be sealed in sterile container, packed in triple layer packing and sent to the testing laboratory. Sample collection should be performed by trained personnel following all the aseptic techniques implemented by WHO.

Transportation of specimens to the Laboratory

Specimen samples should be collected and transported to the appropriate laboratory within 12-24 hrs for confirmatory testing. For safe transport, samples should be packaged in primary, secondary and outer containers and suitable cooling to be maintained using the gel pack. The current regulatory framework proposed by WHO for the transport of infectious substances and patient specimens can be found in 'Guidance on regulations for the transport of infectious substances 2019-2020' applicable from 1st January 2019.

Diagnosis:

Culture method: PLET (polymyxin lysozyme EDTA thallous acetate) medium is a common selective agar medium used for the culture of *B. anthracis*. Colonies appear as circular white to grey-white after overnight incubation at 37°C.

Molecular methods: Traditional Polymerase chain reaction (PCR) and Real time-PCR (RT-PCR) are two molecular diagnostic tools to reconfirm the *B. anthracis* colonies observed in culture test. Template should be prepared using commercially available extraction kit either from genomic DNA or plasmid DNA depending on the target sequence for PCR/ RT-PCR. Appropriate positive and negative control should be always run with test set. If controls give unexpected results, or if an equivocal result is obtained for test data, the cause of the discrepancy should be investigated and the run repeated.

Quality control:

Quality control exercises will be performed with reference laboratories like the National Centre for Disease Control (NCDC), New Delhi for various tests like culture, molecular diagnosis and confirmation of anthrax.
