# Supplementary material for: One Health approach for elimination of human anthrax in a tribal district of Odisha: Study protocol
Source: PLoS One. 2021 May 27;16(5):e0251041. doi: 10.1371/journal.pone.0251041 (PMC8158997; doi:10.1371/journal.pone.0251041)
Supplement: S3 Appendix — (PDF) [file pone.0251041.s003.pdf]

Format for Application for ***Ad-hoc*** Research Projects

and

Guidelines for Operation of Extramural Projects

"One Health" strategy for elimination of human anthrax from an endemic district of Odisha: a demonstration project

Dr. Sanghamitra Pati

Dr. D. Bhattacharya

Dr. Jaya Singh Kshatri

Prof. K. Mishra

Prof. N.Sahu

Dr. A. Padhi

ICMR-Regional Medical Research Centre

Bhubaneswar

Indian Council of Medical Research

V. Ramalingaswami Bhawan, Ansari Nagar, P.Box No. 4911

New Delhi – 110029

Tel. : 26588895, 26588980, 26589794, 26589336 GRAM: SCIENTIFIC

Email: headquarters@icmr.org.in icmrhqds@sansad.nic.in FAX: 011-26588662

INDIAN COUNCIL OF MEDICAL RESEARCH

V. Ramalingawami Bhawan, Ansari Nagar, Post Box Bo. 4911, New Delhi - 110029

APPLICATION FOR GRANT-IN-AID OF AD-HOC RESEARCH PROJECT

(Please furnish 30 copies)

Section A
GENERAL

---

1. Title of the Research Project — **"One Health" strategy for elimination of human anthrax from an endemic district of Odisha: a demonstration project**

2. Name and Designation of

i) Principal Investigator & Email

Dr. Sanghamitra Pati, Scientist-G & Director,
ICMR-Regional Medical Research Centre, Bhubaneswar-751023
drsanghamitra12@gmail.com

ii) Co-Investigator(s) & Email

a. Dr. Debdutta Bhattacharya, Scientist -C
ICMR-Regional Medical Research Centre, Bhubaneswar-751023
drdebdutta.bhattacharya@yahoo.co.in

b. Dr. Jaya Singh Kshatri, Scientist -B (Medical)
ICMR-Regional Medical Research Centre, Bhubaneswar-751023
jsk.icmr@outlook.com

c. Prof. Niranjan Sahoo, Professor Department of Veterinary Medicine, OUAT, nsahoo671@gmail.com

d. Prof. K. Mishra, Prof. and Head, Community Medicine, SSLN Medical College, Koraput; kaushikmishra1965@gmail.com

e. Dr. S.P.Padhi, Addln Director, Public Health, Govt. of Odisha,

f. Dr.Arun Padhi, District Public Health Officer, Koraput

3. Duration of Research Project — 3 years

i) Period which may be needed for collecting the data — 2½ years

ii) Period that may be required for analyzing the data — 6 months

4. Amount of grant-in-aid asked for *(details are to be furnished in Section B)* — Rs. **12668400** (Rupees One crore twenty-six lakhs eight thousand and four hundred only)

Head of A/c		1st year	2nd year	3rd year	Total
**i.**	**Staff**	2340000	2574000	2831400	7745400
**ii.**	**Contingencies**				
	**a. Recurring**	850000	825000	625000	2300000
	**b. Non-recurring (equipment)**	2,00,000	0	0	200000
	**c. Travel**	1,00,000	2,00,000	2,00,000	500000
**iii.**	**Training & workshops**	272000	583000	368000	1223000
**iv.**	**IEC/BCC Activities**	1,00,000	3,00,000	3,00,000	700000
**Total**		**3862000**	**4482000**	**432440**	**12668400**

5. Institution responsible for the research project

**Name**	ICMR-Regional Medical Research Centre, Bhubaneswar
**Postal address**	Chandrasekharpur, Bhubaneswar, Odisha- 751023
**Telephone**	**91-674-2301322**
**Fax**	**91-674-2301351**
**E-mail**	**dir.rmrcb@icmr.gov.in**

**6.**	**Institutional ethical clearance and Project approval** *(Necessary documents indicating institutional ethical clearance must be enclosed for research involving human subjects as also animal experiments).*	Awaited
**7.**	**Is radio tagged material proposed to be used in the project either for clinical trials or experimental purposes?** (*If so, clearance from Nuclear Medicine Committee, Bhabha Atomic Research Centre, Mumbai, indicating should be attached)*	No
**8.**	**Projects involving recombinant DNA/Genetic engineering work should be examined and certificate by the Institutional Biosafety Committee (IBSC) to be enclosed.** *(Guidelines for constitution of IBSC can be obtained from Secretary, Department of Biotechnology, CGO Complex, Lodhi Road, New Delhi-110003.)*	No rDNA/ genetic engineering work involved
**9.**	**Approval of the institutional ethics committee (IEC) should be enclosed.** *Guidelines for IEC for animal experiments should follow CPCSEA requirements and for human studies should follow ICMR guidelines*	IEC approval awaited
**10.**	**The Institution where the study is being done should ensure that there is no financial conflict of interest by the investigators.**	No conflict of interest by investigators

DECLARATION AND ATTESTATION

i. I/We have read the terms and conditions for ICMR Research Grant. All necessary Institutional facilities will be provided if the research project is approved for financial assistance.

ii. I/We agree to submit within one month from the date of termination of the project the final report and a list of articles, both expendable and non-expendable, left on the closure of the project.

iii. I/We agree to submit audited statement of accounts duly audited by the auditors as stipulated by the ICMR.

iv. It is certified that the equipment(s) is/are not available in the Institute/Department or these are available but cannot be spared for the project

v. It is further certified that the equipment(s) required for the project have not been purchased from the funds provided by ICMR for another project(s) in the Institute.

vi. I/We agree to submit (online) all the raw data (along with descriptions) generated from the project to the ICMR Data Repository within one month from the date of completion /termination of the project.

If any equipment already exists with the Department/Institute, the investigator should justify purchase of the another equipment.

Signature of the

a) Principal investigator, Dr.S.Pati ________________________

b) Co-investigator(s), Dr.D.Bhattacharya ________________________

c) Co-investigator(s), Dr.J.S.Kshatri ________________________

d) Co-investigator(s), Dr.K.Mishra ________________________

e) Co-investigator(s), Dr.N.Sahoo ________________________

f) Co-investigator(s), Dr.S.K.Padhi ________________________

g) Co-investigator(s), Dr. A.Padhi ________________________

h) Head of the Department ________________________

Signature of the head of the Institution with seal

Date:

P.S. ICMR should be reminded if no acknowledgement is received within one month from the date of sending the application.

Section - B

DETAILS OF THE RESEARCH PROJECT

Adequate information must be furnished in a brief but self-contained manner to enable the Council to assess the project.

1. Title of the project

"One Health" strategy for elimination of human anthrax from an endemic district of Odisha: a demonstration project

2. Objectives

Aim/Goal

To study the effectiveness of public health interventions based on multi-sectoral strategy following "One Health" approach in reducing the incidence of human anthrax cases to zero in Koraput, an endemic tribal district of Odisha.

Primary objective

- Developing and implementing a package of coordinated interventions using "One-Health" approach to demonstrate a replicable strategy for elimination of animal to human disease transmission of Anthrax in an endemic district of Odisha.

Secondary Objectives

i. Situational analysis regarding disease burden, patterns, knowledge and practices prevalent among the identified stakeholders from the departments of health, animal husbandry, environment/forest and the community.
ii. To develop and implement the following complex of public health intervention packages using the principles of Theory of Change (ToC) framework in the study district
    a. Strengthen the surveillance and tracking systems for early reporting of suspected cases of human and animal anthrax by developing and implementing the "One-Health" Bio-surveillance system.
    b. Establishment of an anthrax diagnosis facility in the state to facilitate early diagnosis and reporting of Human Anthrax cases based on standardized case definitions.
    c. Development of standard operating procedures and human outbreak response protocols for all stakeholder departments in a consultative manner (Health, Animal

resources, Forest and Environment & PRI) and provision of post-exposure prophylaxis of suspected contacts.

d. Capacity building of stakeholders for early detection and appropriate action at district, sub-district, block and village level, including clinical case management and referral, in a standardized and synchronized manner.
e. Create framework for categorization of geographical “Risk-Zones” based on GIS mapping of cases for prioritization of public health interventions such as “ring vaccination” for animals.
f. To develop content and strategy for behavior change by using blended communication approaches for the vulnerable community and other stakeholders.

iii. To evaluate the interventions among the study population and assess the process and outcome on Anthrax burden in the study district along with cost-benefit analysis of interventions.

3. Summary of the proposed research (up to 150 words) indicating overall aims of the research and importance of the research proposal. Application of the work in the context of national priorities of medical research, if any, may also be mentioned.

Anthrax is a major but neglected Zoonotic disease of public health importance in India with Odisha contributing a major share to the disease burden. This project is proposed to study the effectiveness of public health interventions based on multi-sectoral strategy following “One Health” approach in reducing the incidence of human anthrax cases to zero in Koraput, an endemic tribal district of Odisha.

This project would use the principles of Theory of Change to build, implement and study a complex intervention model involving a consultative approach with all stakeholders. We plan to achieve this by establishing a robust surveillance system, building the capacity of all involved stakeholders, supporting early diagnosis of cases, facilitating better inter-departmental co-ordination and creating Risk-Zones using GIS techniques to categorize the interventions within the study area. A strong monitoring and evaluation system would be put in place using logframe analysis.

This study would provide recommendations for policymaking and planning base for future scale up of elimination projects for zoonotic diseases using “One Health” strategies.

4. Present knowledge and relevant bibliography including full titles of articles relating to the project.

Zoonosis is infectious diseases shared by vertebrates and humans in nature. One of the most striking aspects of new, emerging and re-emerging zoonotic diseases has been the unexpected nature of their occurrence. In fact, at least in the developing countries, this can be considered as a major concern calling for a resolution (Kumar et al., 2015). It is widely recognized that zoonotic diseases control and elimination require a joint approach by animal and human health sectors [Yin et al., 2013; WHO, 1970]. Joint surveillance system using One Health approach is the most effective and efficient way of protecting human and animal populations from zoonotic disease like rabies and anthrax in low income countries [Maudlin et al., 2009; Schneider et al., 2005; Chomel et al., 1988]. However, implementation of such surveillance approach in these countries is hampered by several factors: multiple disease challenges, unmotivated One Health workforce, remoteness, lack of appropriate working tools/infrastructure, and low budget, just to mention a few. These factors often led to emergence and spread of zoonotic diseases. Emerging infections usually prove more threatening because we have little information about their origin and many of their epidemiological features remain unknown. The magnitude of their economic impact is also not too well delineated. Zoonotic diseases are of great public health importance in India, where 68% of the workforce relies on farming that is in close contact with domestic animals and poultry with frequent exposure to sick or infected animals. Unhygienic living conditions, lack of education, poor personal hygiene, poor veterinary and public health services, poverty and malnourishment contribute to the dissemination of these zoonoses. Zoonotic diseases are responsible for a large burden on the public health, livestock economies, and wildlife of India (Knobel et al., 2005; Pavani, 2014). In the last couple of decades, array of new infectious agents/diseases depicting animal-human connection were reported and researched across the globe (Kumar et al., 2015). Among these diseases, Anthrax accounts for majority of cases in India (Pavani, 2014).

Anthrax is caused by gram-positive, rod-shaped bacteria known as *Bacillus anthracis*. Anthrax is a worldwide zoonosis that primarily affects herbivorous animals (Spencer 2003). The Anthrax bacterium produces spores which are capable of surviving for many years in the environment. Anthrax most commonly occurs in wild or domesticated warm-blooded grazing animals such as sheep and cattle, but can infect humans causing three types of infections affecting the lungs (pulmonary form), the digestive tract (intestinal form), or the skin (cutaneous form). All types of anthrax can eventually spread throughout the body and cause death if not treated with antibiotics

appropriately at appropriate time. In domestic animals, there is high fatality in cattle, sheep, goats, horses, donkeys and pigs (Turnbull 2008). For wild animals, highest fatalities occur in zebra, antelope, bison, gazelles, impalas, elephants and hippopotami (Turnbull, 2008, Shivachandra, 2016). Omnivores and carnivores are moderately resistant although wild carnivores that scavenge from infected carcasses still succumb to illness (Shivachandra, 2016). Anthrax is considered a seasonal disease as outbreaks tend to follow a prolonged hot and dry period that is followed by heavy rainfall. It is thought that heavy rainfall can carry spores during runoff in clumps of organic matter to concentrate in standing pools or puddles (Hugh-Jones 2009). It is also thought that standing water can move spores upwards into the vegetation as it dries, leaving them in a better position to be ingested by grazing animals (Hugh-Jones 2009). Vultures and wild birds are also a risk for movement of *B. anthracis* to new areas by scavenging infected animal carcasses (Turnbull 2008). It has also been shown that moving animals from an endemic area to a non-endemic area can result in the establishment of the disease in new areas, if the soil conditions are conducive to spore survival (Turnbull 2008).

Importance of anthrax has gained a new dimension because of its application in biological warfare. Japan used the anthrax bug for the first time against Manchuria in 1940s (Harris, 1994). More recently it was death of a Florida photo editor from inhalational anthrax acquired from a letter deliberately contaminated with spores of Bacillus anthracis thrust this extremely rare infection in USA into public awareness. Between September 18, 2001 and November 21, 2001, there were13 cases of cutaneous anthrax and 11 cases of inhalational anthrax in association with known or presumed exposure to Anthrax spores in contaminated mail in USA. World was forced to recognize the possibility that anthrax may be used as a biologic weapon in 1979, when at least 66 people in Sverdlovsk died in the largest known epidemic of inhalational anthrax. This epidemic followed the accidental release of anthrax spores into the atmosphere by a research facility involved in “weaponizing” anthrax by preparing finely milled, non-clumping (electrostatically neutral) spores that are optimal for dissemination and inhalation and that produce toxins when they germinate (Swartz, 2001).

After the Gulf War, Iraq admitted producing and deploying such weaponized anthrax in missiles; thus a clear threat remains. Though the disease has been almost totally eradicated from Western world, the disease - in real sense - has never been fully contained in Asian, African and Central American countries, as the livestock in these countries are only marginally subjected to veterinary control and the environmental conditions here favour an animal-soil-animal transmission. India, also, is an endemic region for animal anthrax because of unprotected

livestock population (Bhat et al., 1989).It is this which gives rise to emergence of human anthrax from time to time in some parts of the country (Dutta et al., 2011).

Much has been written and hypothesized about the effects of season, rainfall, temperature, soil, vegetation, host condition and population density on the epidemiology of anthrax, but little agreement exists on the roles played by these factors in the incidence of the disease, and this is a topic in need of further research (WHO, 2008). *B. anthracis* appears to be one of the most monomorphic species known, i.e. isolates from whatever type of source or geographical location are almost identical phenotypically and genotypically. Phenotypically, strain differences are only apparent in nonquantifiable or semi quantifiable characteristics, such as colonial morphology, flocculation in broth culture, cell size, multiplication rate, sporulation efficiency, LD50in animal tests, etc. The genetic basis for these differences has not been established and at the molecular level genomic differences long proved difficult to detect. The biochemical, serological or phage typing methods available in the case of other pathogens have proved of no value for identifying different strains of *B. anthracis*.

In the past five years important progress has been achieved in determining phylogenetic relationships among isolates worldwide, through multilocus variable number tandem repeat (VNTR) analysis (MLVA)(Keim et al., 2000; Keim & Smith, 2002). MLVA examines a number of DNA segments within the chromosome and plasmids of *B. anthracis* for the presence of strain-dependent patterns of repeated specific nucleotide sequences and has enabled a broad separation of isolates into two major clonal groups, A and B, with four or more minor clusters in the Abranch and two minor clusters in the B branch. The A branch is the most common worldwide, with theB1 branch found only in southern Africa and B2 scattered worldwide outside Africa (Keim & Smith, 2002).

National status:

Anthrax is most common in agricultural regions of Central and South America, Sub-Saharan Africa, Central and South-western Asia, Southern and Eastern Europe, and the Caribbean. In India anthrax is enzootic in states like Andhra Pradesh, Jammu and Kashmir, Tamil Nadu, Odisha and Karnataka (NCDC, 2005). The actual incidence of anthrax in India is not known accurately due to the fact that a large number of cases go unreported and only a fraction of human cases receive medical attention in a hospital. Cases treated on site in a village are hardly brought tothe notice of authorities. Hence, the incidence of anthrax in man is likely to be higher than reported in literature (Balachandrudu et al., 2018).

During last 15 years out of 30 revenue districts in Odisha, 14 districts have witnessed outbreaks of anthrax affecting at least 1208 people of which 436 had died (Patil 2010 and IDSP, Odisha 2016). The anthrax outbreaks are an annual phenomenon in the state and the most frequently affected districts are Koraput, Rayagada, Malkangiri, Sundergarh, Deogarh, Anugul and Kandhamal, whereas the occurrence of anthrax in other districts such as Nawrangpur, Nuapada, Mayurbhanj, Baragarh, Sambalpur, Bolangir, Nayagarh and Cuttack are recorded at long intervals. From the available information, it has been observed that very often the tribal communities those who eat or handle carcass of dead animals of these districts have been affected. Among the animal cattle, buffalo, sheep, goat and pigs are most susceptible animals and the disease is seen in the state round the year in the aforesaid animals. The morbidity rate due to anthrax in animals was 1.45% and the mortality rate was 12.56 % with case fatality rate of 30.20 % and spread over 19 districts in the state. Herbivores, the primary hosts of this pathogen, are usually infected anthrax by ingestion of spores while grazing or browsing. Human infection was usually a result of contacting ill animals during agricultural activities or processing contaminated animal products. Limited person-to-person transmission has been reported. As majority of the tribal areas have poor public health infrastructure, it makes the perfect amalgamation and interface of risk factors that are conducive for zoonotic transmission of anthrax to the human population. Hence the probability of occurrence of anthrax outbreak in the human population would increase on logarithmic scale, each time an episode of anthrax detected in animal population.

From vulnerability point of view Odisha is highly prone to risk of zoonotic pathogen specially Anthrax outbreaks in the human population in the coming years because the state has very high concentration of tribal population (>22 %) who depend less on agriculture and more on forest and animal produce for food because of illiteracy and poverty. Hence situation specific appropriate strategies can be developed to prevent the spread of outbreak of the disease.

Among the districts, that have reported Human Anthrax in Odisha, Koraput districts tops the list with more than 300 human cases and more than 10 deaths with confirmed Anthrax infection during the last 6 years. During July, 2018, three deaths and 10 cases were reported form Laxmipur block of Koraput district. All the cases were from a group of tribal people engaged in deskinning and butchering of a dead animal. Therefore the present study is proposed to be undertaken in the district of Koraput which can be replicated in other districts too in later stage by the state government.

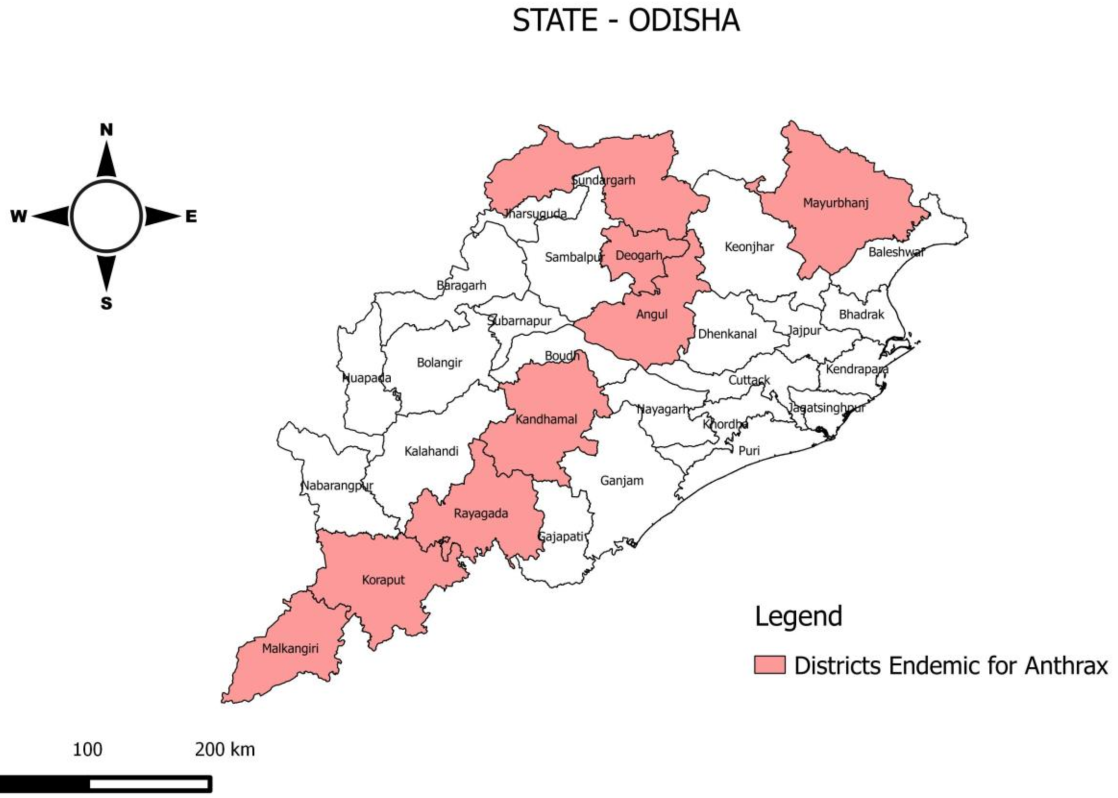

S Fig 1: Map of Odisha showing (red colour) districts that have experienced outbreaks of anthrax during 2010-2016.

References

1. SP Singh, R. (2015). Overview of Emerging Zoonoses in India: Areas of Concern. *Journal of Tropical Diseases*, 03(03).
2. Zhou XN. Prioritizing research for “One health - One world”. Infect Dis Poverty; 2012; 1 (1)
3. Yin W, Dong J, Tu C et al. Challenges and needs for china to eliminate rabies, Infectious Diseases of Poverty; 2013; 2(1)
4. I. Maudlin, M. C. Eisler, and S. C. Welburn. Neglected and endemic zoonoses. Philosophical Transactions of the Royal Society B: Biological Sciences;2009; 364 (1530); 2777–2787
5. WHO/DFID-AHP, FAO, OIE. The control of neglected zoonotic diseases: A route to poverty alleviation; Geneva, 2006.
6. Hopkins DR: Disease eradication. N Engl J Med. 2013, 368: 54-63.
7. Ernest T, Lin A, Xia Z, et al. Infectious Diseases of Poverty. Biomed Central; 2014 **3**:17 https://doi.org/10.1186/2049-9957-3-17

8. Utzinger J, Raso G, Brooker S, de Savigny D, Tanner M, Ørnbjerg N, Singer BH, N'Goran EK: Schistosomiasis and neglected tropical diseases: towards integrated and sustainable control and a word of caution. Parasitology. 2009, 136: 1859-1874.
9. Hotez PJ, Molyneux DH, Fenwick A, Kumaresan J, Ehrlich Sachs S, Sachs JD, Savioli L: Control of neglected tropical diseases. N Engl J Med. 2007, 357: 1018-1027.
10. Bhattacharya S: Reflections on the eradication of smallpox. Lancet. 2010, 375: 1602-1603.
11. Larson HJ, Ghinai I: Lessons from polio eradication. Nature. 2011, 473: 446-447.
12. Morse SS, Mazet JA, Woolhouse M, Parrish CR, Carroll D, Karesh WB, Zambrana-Torrelio C, Lipkin WI, Daszak P: Prediction and prevention of the next pandemic zoonosis. Lancet. 2012, 380: 1956-1965.
13. Zhang H, Lai S, Wang L, Zhao D, Zhou D, Lan Y, Buckeridge DL, Li Z, Yang W: Improving the performance of outbreak detection algorithms by classifying the levels of disease incidence. PLoS One. 2013, 8: e71803
14. Moonen B, Cohen JM, Snow RW, Slutsker L, Drakeley C, Smith DL, Abeyasinghe RR, Rodriguez MH, Maharaj R, Tanner M, Targett G: Operational strategies to achieve and maintain malaria elimination. Lancet. 2010, 376: 1592-1603.
15. Bousema T, Griffin JT, Sauerwein RW, Smith DL, Churcher TS, Takken W, Ghani A, Drakley C, Gosling RD: Hitting hotspots: spatial targeting of malaria for control and elimination. PLoS Med. 2012, 9: e1001165
16. Utzinger J: A research and development agenda for the control and elimination of human helminthiases. PLoSNegl Trop Dis. 2012, 6: e1646
17. Wu XH, Wang XH, Utzinger J, Yang K, Kristensen TK, Bergsquist R, Zhao GM, Dang H, Zhou XN: Spatio-temporal correlation between human and bovine schistosomiasis in China: insight from three national sampling surveys. Geospat Health. 2007, 2: 75-84.
18. Tan CG, Sandhu HS, Crawford DC, Redd SC, Beach MJ, Buehler JW, Bresnitz EA, Pinner RW, Bell BP, Regional Anthrax Surveillance Team: Surveillance for anthrax cases associated with contaminated letters, New Jersey, Delaware, and Pennsylvania, 2001. EmergInf Dis. 2002, 8: 1073-1077.
19. WHO: Anthrax in humans and animals – 4th ed. ISBN 978 92 4 154753 6; WHO, Geneva-2008
20. Maini R, Mounier-Jack S, Borghi J. How to and how not to develop a theory of change to evaluate a complex intervention: reflections on an experience in the Democratic Republic of Congo. BMJ Glob Health 2018;3:e000617. doi:10.1136/ bmjgh-2017-000617

5. Preliminary work already done by the Investigator on this problem, e.g. selection of subjects, standardization of methods, with results, if any.

A preliminary study has been being carried out in 4 endemic districts of Odisha (Koraput, Rayagada, Sundergarh and Malkangiri) for Anthrax to understand the knowledge, attitude & practice of the population effecting management of Anthrax in these region.

- 557 households covered in 4 endemic districts.
- 49 IDIs were conducted with officials representing various stake holders.
- 11 FGDs were also conducted.

Highlights of finding

- 20% of the respondents never heard about Anthrax disease.
- 48% of respondents were unaware about the mode of transmission.
- About 18% of the respondents were found to consume dead animals.
- Butchering and deskinning of dead animal accounted for majority (36%) of the Anthrax cases (88) interviewed.

Roadblocks identified for Anthrax Control: Gap Analysis

a) Lack of awareness and stigma about Anthrax in villagers
b) Lack of interdepartmental co-ordination and man power
c) Behaviour and food practices of people

6. **Links with other ICMR projects (ad-hoc, task force or collaborative):** None

7. List of important publications of last 5 years of the all the investigators in the relevant fields (enclose reprints, if available)

i. **Pati S**. Nations within a nation: variations in epidemiological transition across the states of India, 1990-2016 in the Global Burden of Disease Study India & State-level Disease Burden Initiative Collaborators.. ***Lancet.*** **2017**; S0140-6736(17)32804-0. doi: 10.1016/S0140-6736(17)32804-0.

ii. S Pati, AS Chauhan, S Mahapatra, MR Sinha, **S Pati**. Practicing health promotion in primary care–a reflective enquiry. ***Journal of Preventive Medicine and Hygiene*** **2017**; 58 (4), 288.

iii. BP Nunes, ADP Chiavegatto Filho, **S Pati**, DSC Teixeira, TR Flores, et al. Contextual and individual inequalities of multimorbidity in Brazilian adults: a cross-sectional national-based study. ***BMJ open***. **2017**; 7 (6), e015885.

iv. Swain S, **Pati S,** Pati S. A chart review of morbidity patterns among adult patients attending primary care setting in urban Odisha, India: An International Classification of Primary Care experience. ***J Family Med Prim Care***. **2017;** 6(2):316-322. doi: 10.4103/2249-4863.220029.

v. Gustavo Dias Ferreira, Jose Augusto Simo, Chamara Senaratna, **Sanghamitra Pati**, Pierre Fernando Timm, Sandro Rodrigues Batista, Bruno Pereira Nunes. Physiological markers and multimorbidity: A systematic review. Journal of Comorbidity 2018; 8: 1-10.

vi. Chandar B, Pati S, **Bhattacharya D**. An overview of New Delhi Metallo-βeta lactamase-1 and Extended Spectrum Beta Lactamase producing bacteria: need for an alternate. J Antimicrob Agents 2018, 4(1):155. DOI: 10.4172/2472-1212.1000155.

vii. **Bhattacharya** D, Dey S, Pazhani GP, Ramamurthy T, Parande MV, Kholkute SD, Roy S. Vibrio cholerae O1 El Tor variant and emergence of Haitian ctxB variant in the strains isolated from South India. Med Microbiol & Immunol 2016; 205(2): 195-200.

viii. Purwar S, **Bhattacharya D**, Metgud SC, Kumar D, Chitambar SD, Roy S. A cross-sectional study on etiology of diarrhoeal disease, India. Indian J Med Microbiol 2016; 34(3):375-9.

ix. **Bhattacharya D**, Bhattacharya H, Sayi DS, Bharadwaj AP, Singhania M, Sugunan AP, Roy S. Changing patterns and widening of antibiotic resistance in Shigella sp. over a decade (2000-2011), Andaman Islands, India. Epidemiol Infect 2015; 143(3): 470-77.

x. India State-Level Disease Burden Initiative Diabetes Collaborators. The increasing burden of diabetes and variations among the states of India: the Global Burden of Disease Study 1990–2016. Lancet Glob Health 2018. 1-11.

xi. Pradhan N, Pradhan P, **Pati S and** Hazra RK . Epidemiological profile of malaria among various socio-demographic groups in a western district of Odisha, India.. Infez Med. 2018, 26(1):37-45.

xii. Tripathy RM, **Kshatri JS,** Sovani V. Epidemiology of animal bites in Berhampur, Odisha, and safety of Rabies immune globulin. Journal of APCRI, 2017; 18 (3) ;18-21

xiii. **Kshatri JS,** Karmee N, Patro SK, Tripathy RM. Patient satisfaction and out-patient services assessment in a tertiary hospital of South Odisha, India. *Int J Adv Med* 2017;4:497-501.

8. Detailed research plan. (Give here the design of the study, indicating the total number of cases/samples/animals to be studied, the mode of selection of subjects specially in experiments involving human beings, equipments and other materials to be used, methodology/techniques to be employed for evaluating the results including statistical methods any potential to obtain patents etc.)

A. **Design-** Cross Sectional study design (for baseline and end line study). This would be demonstration study of effectiveness of structured public health interventions to eliminate human Anthrax from a specified district by a comparative analysis of baseline and end line data.

B. Study Setting: Based on the data on human and animal related cases available with IDSP, State veterinary departments, 8 districts has been identified as high risk zone for Anthrax. One representative and endemic district will be taken up for the demonstration. For this purpose, Koraput, a tribal district endemic for Anthrax has been selected. It is situated in

south Odisha spread out over 8807 sq km in the Eastern Ghats with 14 blocks and 2028 revenue villages.

C. **Study Participants-**The study participants for the baseline and end line study will be sampled from among the stakeholders identified in the district. As this project would use theory of change, a comprehensive stakeholder mapping and analysis will be carried out based on the following broad categories. Additionally, participants for the qualitative study will be selected from known hot-spots of outbreaks and tribal groups with practices making them vulnerable for infection acquisition and propagation.

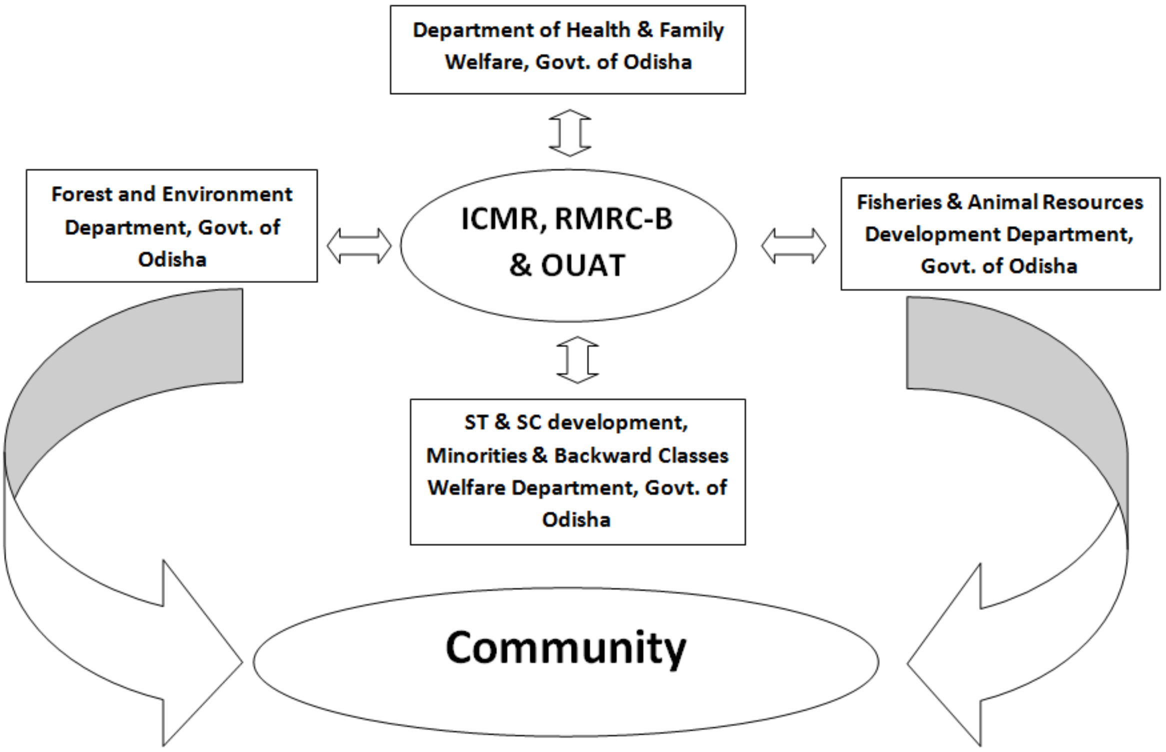

Role of various stakeholders in the Zoonosis coordination working group

ACE- Aims	Zoonosis coordination working group, Odisha					
	ICMR-RMRC Bhubaneswar	OUAT, Bhubaneswar	Dept H&FW, Odisha	Dept. of Forest & Environment, Odisha	Dept. of Fisheris & Animal resource development	Dept. of SC & ST development, Odisha
**1. Diagnostics for Anthrax in humans**						
**a.** Establishment of laboratory facility for diagnosis of human Anthrax by culture & molecular techniques	×					
**b.** Establishment of standard protocols and proficiency testing panels for Anthrax diagnosis	×					
**2. Diagnostics for Anthrax in livestock, wildlife and the environment in Odisha**						
**a.** Establishment of laboratory facility for diagnosis of Anthrax in livestock, wildlife and the environment		×				
**3. *Surveillance, reporting, molecular epidemiology* and *risk zone identification* of Anthrax in humans, livestock, wildlife and the environment in India**						
**a.** Establishment of a surveillance mechanism of Human Anthrax in Koraput	×	×	×			×
**b.** Establishment of a surveillance mechanism of Anthrax in livestock, wildlife and the environment of Koraput			×	×	×	
**c.** Molecular epidemiology of the circulating strains of *B. anthracis* in Odisha	×	×				
**d.** Identification of risk zone for Anthrax by GIS mapping using retrospective and prospective data	×	×	×	×	×	
**3. Situational analysis regarding disease burden, patterns, knowledge and practices prevalent among the identified stakeholders**						
**a.** Collating information on vaccine coverage and vaccination strategies in target livestock species and in high-risk livestock production systems		×			×	
**b.** Practices for anthrax outbreak (detection, reporting, disposal, disinfection, treatment/prophylaxis, and education) and in humans, livestock, and wildlife in endemic settings	×	×	×	×	×	×
**4. Development of SOP and capacity building for surveillance, outbreak investigation, laboratory capacity, vaccination, and risk communication for the control and eventual elimination of Anthrax from Koraput district**						
**a.** Evaluation of existing capabilities and barriers for anthrax surveillance and control in Koraput	×	×	×	×	×	×
**b.** Development of standard operating procedures and outbreak response protocols for all stakeholder departments in a consultative manner	×	×	×	×	×	×
**c.** Establishing training programs for key stakeholders	×	×	×	×	×	×
**d.** Development and implementation of strategies for community education and risk communication	×	×	×	×	×	×

Health Care Sector	Animal Care Sector	Community
Clinical service providers	Veterinary doctors	Service utilizer clients
Program managers	Livestock inspectors	PRI members
Health workers	Forest guards	NGO and SHGs
ASHA workers		Cattle owners/grazers
		Hunter/gatherers
		Village reisdents

D. <u>Sampling:</u>

Multi stage random sampling with strata for each stakeholder group will be used for data collection in the baseline and end line study. Interventions will be planned and implemented for the entire district & its 14 blocks. Qualitative study will be carried out on a sub set of the sampled stakeholders selected by systematic random sampling. Additionally, for the qualitative part, snowball sampling will be used to identify and track suspected cases and contacts in the recent past.

E. **<u>Sample size:</u>**

The sample size required for the quantitative component of baseline (and end line) study was estimated to be 4015. For uniformity of sampling, this was rounded off to 4060 in total.

This was calculated by the following formula:

$$Minimum\ Sample\ Size\ required = \frac{[\ (1.96)^2 * P * (1-P)]}{d^2} * \text{(Design Effect)}$$

Where,

Level of confidence assumed at 95%, corresponding to Z=1.96;

P= Prevalence of exposure to anthrax (Assumed at 5%)

d= Relative precision at 20% of P

Design effect = 2.

Non Response rate = 10%

For the qualitative component, data collection would continue till saturation is reached based on principle of maximum divergence.

F. **<u>Mode of selection of subjects:</u>**

Subjects will be selected for the quantitative study from within each strata of stakeholders by multi stage random sampling. Qualitative study will be carried out on a sub set of the sampled stakeholders selected by systematic random sampling. For eg. the following mode of selection for community members would be used to select subjects (4060) from 70 villages of the 14 blocks in Koraput:

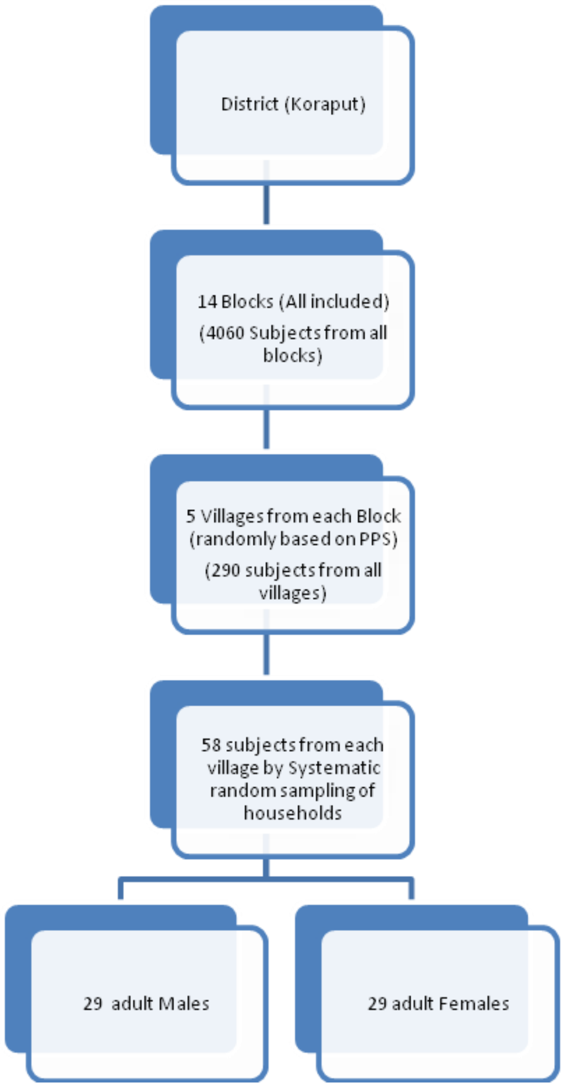

G. **<u>Methodology-</u>**

The study would test the effectiveness of a complex of public health intervention packages developed using the ToC framework for elimination of human anthrax in Koraput district of Odisha by comparative analysis of baseline and end line data.

Baseline Study

A baseline study and a desk review including analysis of health system preparedness would be carried out prior to roll out of the interventions using a mixed method approach for data collection on quantitative and qualitative indicators of disease burden, knowledge and practices. Human Resource mapping in the district for relevant departments would be done. This would also use GIS analysis to identify frequent hot-spots of outbreaks for targeted data collection for the qualitative study. Special focus would also be given to study tribal practices which contribute to acquiring infection and disease transmission. Quantitative data will be obtained from the sample population and stakeholders using a pre tested structured questionnaire.

Focus group discussions (8-10) for each of the stakeholder and vulnerable groups will be conducted along with key informant interviews and in depth interviews for collection of qualitative data. Disease burden will be assessed by reviewing hospital records of all Community Health Centres, District HQ hospital and Medical College Hospital. Qualitative data from suspected & confirmed past cases and contacts will be collected by using snowball sampling in villages.

Public Health Interventions

This study will use the principles of Theory of Change (ToC) to build and test the complex intervention model for the said objectives. The construction of a ToC typically occurs through a consultative process, requiring stakeholders to reflect on how their programmes can bring about change. ToCs help make explicit any underlying assumptions, acknowledge the role of context and provide evidence to justify the chain of causal pathways. The following interventions will be developed and tested:

- ✓ Strengthening the health care system and surveillance system for early reporting of suspected cases of human and animal anthrax by developing and implementing the "One-Health" Bio-surveillance system.

- ✓ Establishment of an anthrax diagnosis facility in the state to facilitate early diagnosis and reporting of anthrax cases based on standardized case definitions.
- ✓ Operationalization of standard operating procedures and outbreak response protocols for all stakeholder departments in a consultative manner
- ✓ Provision of post-exposure prophylaxis of suspected contacts by the health department at the health facility and community.
- ✓ Livestock vaccination program by the state on a large scale and strategy of "ring vaccination" during outbreaks followed by a cost-benefit analysis of the same.
- ✓ Placing a framework for categorization of geographical "Risk-Zones" based on GIS mapping of cases for prioritization of public health interventions.
- ✓ Capacity building of stakeholders for early detection and appropriate action at district, sub-district, block and village level, including clinical case management and referral.
- ✓ Targeted and generalized round the year IEC/BCC activities to be implemented by the state departments in a coordinated manner.

The interventions would be implemented in the entire district in partnership with various departments of the state (Health, Animal resources, Forest and Environment & PRI) in the following manner:

- o Developing a "Risk zoning" protocol for the district using real time data and GIS mapping to zone the geographical limits of the district into a RED (Action), YELLOW (Alert) and GREEN (Monitor) zones during and following an outbreak. Capacity building, Surveillance and IEC/BCC activities shall depend on the prioritization zone of the location.
- o Advocacy for a strong free of cost routine and outbreak vaccination program in the district using findings of the cost-benefit analysis, desk review and baseline study.
- o Development of departmental and inter departmental Standard Operating Procedures for both exclusive and inter-operable activities and conduct of multi departmental co-ordination meetings as per the agreed upon calendar
- o Development of training and capacity building material for each stakeholder group, finalization of training calendar and microplan followed by conduct of Trainings for capacity building among stakeholders from the health care sector and animal care sector as given in the table above.

- Development of IEC material for each group of stakeholders and assisting the state in carrying out IEC and BCC activities in the community.
- Strengthening of surveillance for anthrax by developing surveillance protocols by adaptation of WHO protocols, testing and implementing the surveillance system and facilitating a regional diagnostics service for anthrax as per the following case definitions and diagnostics protocol:

B. anthracis case definitions

Suspect: A case that meets the clinical criteria, but with no presumptive or confirmatory laboratory evidence, or epidemiologic evidence relating it to anthrax

Probable: A case that meets the clinical criteria AND has presumptive laboratory test results

Confirmed: A case that meets the clinical criteria AND has confirmatory laboratory test results

The testing algorithm for *B.anthracis*is provided below.

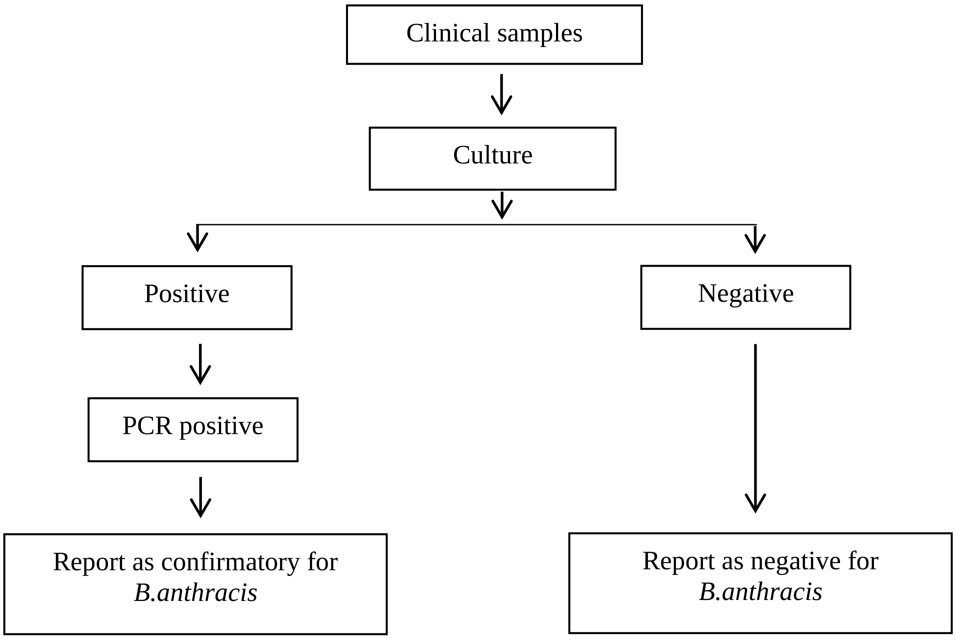

Sample collection:

Swab: Samples will be collected using sterile swabs in duplicate (for culture& PCR) from

a) Vesicular Lesions

b) Eschers
c) Ulcers

The swabs will be sealed in tube, packed in triple layer packing and sent to the testing laboratory.

All the collection of samples will be performed by trained personnel using appropriate PPE.

Transporting/Shipping Specimens to the Laboratory

Specimen samples will be collected and transported to the appropriate laboratory with 12-24hrs for confirmatory testing. For safe transport, samples will be packaged in primary, secondary and outer containers and suitable cooling will be maintained using gel pack.

Diagnosis:

Culture method: Samples will be inoculated into PLET (polymyxin lysozyme EDTA thallous acetate) media and incubated overnight at 37^{0}C. *B.anthacis* will appear as circular creamy-white to grey white colonies.

Molecular methods: The sample will be subjected to DNA isolation using QIAamp DNA mini kit (Qiagen).

PCR: The DNA will be subjected to PCR based amplification of gene coding for protective antigen (597bp) and capsule (873bp). 5 µl of extracted DNA from clinical samples will be used as template. The PCR mixture (total volume, 50 µl) included 37.75 µl of sterile MilliQ water, 5.0 µl of 10X PCR buffer (Bangalore Genei, India), 1.0 µl of 10 mMdNTP, 0.25 µl of Taq DNA polymerase (Bangalore Genei, India), and 1 µl of 20 µl of each primer. DNA amplification will be carried out in a PCR machine by using the following conditions: 5 min at 95°C, followed by 30 cycles of 95°C for 30 s, 55°C for 30 s, and 72°C for 30s. Following primers will be used for conventional PCR

Primer Sequences -

1. Protective Antigen:

a. PA 5 3048-3029: TCC TAA CAC TAA CGA AGT CG

b. PA 8 2452-2471: GAG GTA GAA GGA TAT ACG GT

2. Capsule:

a. 1234 1411-1430: CTG AGC CAT TAA TCG ATA TG

b. 1301 2257-2283: TCC CAC TTA CGT AAT CTG AG

rt-PCR: The colonies typical of B.anthracis will be subjected to rt-PCR by standard protocol (Ellerbrok et al., 2002). The primers and probe to be used is listed below

1. Protective Antigen (PA):

a) PA-Forward: CGG ATC AAG TAT ATG GGA ATA TAG CAA
b) PA-Reverse: CCG GTT TAG TCG TTT CTA ATG GAT
c) PA-Probe: FAM-CTC GAA CTG GAG TGA AGT GTT ACC GCA AAT-BHQ1

2. Capsule (Cap):

a) Cap-Forward: ACG TAT GGT GTT TCA AGA TTC ATG
b) Cap-Reverse: ATT TTC GTC TCA TTC TAC CTC ACC
c) Cap-Probe: FAM-CCA CGG AAT TCA AAA ATC TCA AAT GGC AT-BHQ1

3. RNase P (RP):

a) RP-Forward: AGA TTT GGA CCT GCG AGC G
b) RP-Reverse: GAG CGG CTG TCT CCA CAA GT
c) RP-Probe: FAM-TTC TGA CCT GAA GGC TCT GCG CG-BHQ1

Interpretation of real time-PCR data

If controls give unexpected results, or if an equivocal result is obtained for test data, the cause of the discrepancy should be investigated and the run repeated.

1. Controls

a) *B. anthracis* positive control DNA reactions— should exhibit growth curves within 40 cycles for PA and Cap (FAM dye). All reactions should exhibit growth curves within 40 cycles for the IPC (VIC dye).
b) b. RNAse P positive control DNA reactions—should exhibit growth curves within 40 cycles for RP (FAM dye) and IPC (VIC dye).
c) c. NTC reactions—should not exhibit growth curves within 40 cycles for PA, Cap, and RP (FAM dye). All reactions should exhibit growth curves within 40 cycles for the IPC (VIC dye).
d) d. EB reactions—should not exhibit growth curves within 40 cycles for PA, Cap, and RP (FAM dye). All reactions should exhibit growth curves within 40 cycles for the IPC (VIC dye).

2. Test data (clinical specimen DNA)

a) Positive—if PA, Cap, and RP (FAM dye), and IPC (VIC dye) all exhibit growth curves within 40 cycles
b) Equivocal—if either PA or Cap (FAM dye) exhibits growth curves within 40 cycles, but not both. RP (FAM dye) and IPC (VIC dye) should still exhibit growth curves within 40 cycles.
c) Negative—both PA and Cap (FAM dye) do not exhibit growth curves within 40 cycles. RP (FAM) and IPC (VIC dye) should still exhibit growth curves within 40 cycles.

Quality control:

Quality control exercises will be performed with reference laboratories like National Centre for Disease Control (NCDC), New Delhi for various tests like culture & PCR diagnosis & confirmation of Anthrax.

Implementation plan:

We would use a team approach, with external and domain expert representations, to carry out the activities of the project. The following standing committees / teams with pre defined clear cut roles and responsibilities will be constituted by the investigators for the duration of the study-

- Technical Advisory Team
- Integrated Surveillance Team
- Multi-departmental co-ordination team
- Capacity Building and training team
- Monitoring & Evaluation team

The following table provides the proposed constituents of each team.

Table 1. Project teams and the constituents

**Team Name**	**Team members**
Project Management Team	PI, Co-PIs, External Expert Advisor
Monitoring and Evaluation Team	Scientist-C (Public Health) , Research Assistant, External Expert (Veterinary), External Expert (Health System)
Technical Advisory Team	PI, Co-PIs, Domain Experts (1 Veterinarian, 1 Physician, 1 Public health specialist, 1 Microbiologist), Sc. C (Public Health) Sc. B (Non-medical)
Capacity Building and Training Team	Co-PIs, Research Assistant, Faculty members from partner agencies, Sc. C (Public Health) Sc. B (Non-medical)

Multi-Departmental Co-Ordination Team	Research Assistant, Sc. C (Public Health) Sc. B (Non-medical
Integrated Surveillance Team	Co-PI, Research Assistant, Field Investigators, Data Entry Operators, External experts (1 Bio surveillance, 1 Public Health surveillance)

Team Meetings- To carry out the activities specified, the constituted teams shall meet as per the following calendar-

- ✓ PMT- As and when required (6 meetings in total , approx)
- ✓ MET- Quarterly (12 in total)
- ✓ TAT - Monthly for the first 6 months; quarterly for the next 6 months; then biannually till completion. (12 in total)
- ✓ CBT- as required
- ✓ MDC- Quarterly (12 in total)
- ✓ IST- Biannually (6 in total)

For robust implementation and monitoring, the project interventions are sub-categorized into activities with the pre-constituted teams having defined roles for execution of the same. The project activities and teams responsible is demonstrated in the table below-

Table 2. ***Project Activities and Team responsible***

**Activity**	**Team responsible**	
Formation of study teams	Project Management Team	***Monitoring & evaluation***
Development of surveillance protocols	Technical advisory team,<br>Integrated surveillance team	
Testing and implementing a surveillance system	Integrated surveillance team	
Development of SOPs	Technical advisory team, MDC team	

Development of IEC materials	Technical advisory team	
Carrying out IEC/BCC campaign	Project Management Team	
Development of Training & Capacity Building materials	Technical advisory team, CB & training teams	
Conduct of Trainings	CB & training teams	
Finalization of training calendar	Project Management Team, CB & training teams	
Calendar of MDC meetings	Project Management Team, MDC team	
Conduct of MDC meetings	MDC team	
Risk Zoning	Project Management Team	

Trainings for capacity building- Based on the HR of the district, 1 training each for program managers, data handlers, and animal handlers, 2 for medical officers and 5 batches for sub-centre workers will be carried out in phases. 4 trainings for ASHA Supervisors will be carried out and subsequent capacity building of ASHA workers and dissemination of the SOPs for them will be carried out in conjunction with regular NHM activities and in partnership with the already trained staff using a cascade model.

IEC/BCC activities-These activities will be carried out as per the recommendations of the TAT round the year using the regular system channels like health centres and public places for dissemination. A mass media campaign will also be carried out in an intensive manner. These scope, duration and intensity of these activities shall depend on the risk-zone of a particular region.

The timeline of the project is given below:

Table 3. Gantt Chart of the Project

**Sl. No.**	**Activity**	**Year-1**				**Year-2**				**Year-3**			
		Quarter				Quarter				Quarter			
		1	2	3	4	1	2	3	4	1	2	3	4
1.	Recruitment of staff and MOUs with partner agencies												
2.	Baseline study & gap analysis												
3.	Formation of study teams												
4.	Development of surveillance protocols												
5.	Establishment and operationalization of state level anthrax diagnostic facility												
6.	Testing and implementing a surveillance system												
7.	Development of SOPs												
8.	Development of IEC materials												
9.	Carrying out IEC/BCC campaign												
10.	Development of Training & Capacity Building materials												
11.	Finalization of training calendar												
12.	Conduct of Trainings												
13.	Calendar of MDC meetings												
14.	Conduct of MDC meetings												
15.	Risk Zoning												
16.	End Line study												
17.	Report writing, Submission and Dissemination												

H. **<u>Monitoring & Evaluation plan</u>**- Monitoring and evaluation mechanisms shall be carried out by the monitoring and evaluation team constituted by the PMT and including external members. They may use the following LogFrame as reference for monitoring of the project indicators during implementation of the interventions. Evaluation would be done by comparison between baseline and end line data. Mid-term evaluation would be done as and when deemed necessary.

Table 4. Project Logic Model

Anthrax Elimination from Humans is achieved in a geographical area by a robust surveillance system reporting 0 cases of Human Anthrax for at least 3 consecutive years

INPUTS	ACTIVITIES	OUTPUTS	INDICATORS	OUTCOMES	IMPACT
Grant	Development, Testing and implementing a surveillance system	Robust Surveillance for Human & animal Anthrax	Monthly "0 reporting" of human cases, Monthly reporting of animal cases with at least 1 positive case of animal anthrax annually	No transmission of Anthrax from animals to Humans.	Elimination of Anthrax leading to improved Health in general for the endemic states
Community	Advocacy for livestock vaccination programs	Livestock vaccination program	Improved vaccination coverage among livestock (>50%)	Decreased disease incidence among animals	
	Development of SOPs for inter departmental co-ordination	Improved Inter-departmental Co-Ordination and response	Nomination of nodal persons from each dept& Establishment of Lines of communication between them.	Reduction of Human Anthrax Cases to ZERO.	
Multi-Level Partnerships with stakeholders	Carrying out IEC/BCC campaign	Improved Awareness in the community regarding Anthrax	Significant improvement in KAP as compared to baseline survey	A Sustainable Surveillance model for Zoonotic Diseases.	Elimination programs initiated for other Zoonotic diseases
HR/Staff	Trainings and capacity building of stakeholders	Improved Knowledge of all stakeholders involved	Improved Pre-Test Vs. Post Test scores along with Mid-term Knowledge assessment scores	A replicable strategy that may be modified for elimination programs for other zoonotic diseases.	

Inter agency Convergence	Calendar & Conduct of MDC meetings	Regular MDC Meetings for removal of bottlenecks	Quarterly MDC Meetings documented with minutes shared with Policy makers	Risk Zones of Zoonotic Diseases based on GIS and Surveillance data	Reduced disease and economic Burden on the population
Expert feedback	Risk Zoning	A geographical Risk Zone delineated for activities of Control and Elimination	Creation and review of GIS data on an annual basis.		
Information Technology aids	Data analysis	A Comprehensive and replicable Strategy for Elimination of Anthrax from endemic districts is made available	Report shared with Stakeholders and policy makers by a dissemination workshop and a technical CME		
Baseline data, Teams	Reporting				

I. **Statistical plan-**Descriptive statistics will be used for analysis of quantitative data and qualitative data will be analysed by social mapping, thematic analysis and network diagrams. Trainings effectiveness in enhancing knowledge will be assessed by structured pre- and post-course assessments. Comparison of baseline and end line data will be made by applying tests of statistical significance where applicable with a level of significance established at p=0.05. A Bottleneck analysis using appropriate models will be done for the surveillance system in place.

J. Patents-NA

9. Facilities in terms of equipment, etc, available at the sponsoring institution for the proposed investigation.

a) BSL3 Laboratory
b) Biosafety cabinet type II
c) Real Time PCR machine
d) PCR machine
e) Gel Electrophoresis apparatus
f) Shaker Incubator
g) Hot air oven
h) Autoclave
i) Gel documentation system
j) Fluorescent and compound Microscope
k) Central Instrumentation room
l) Dedicated laboratory

10. Budget requirements (with detailed break-up and full justification):

Sl. No	Head of A/c	1st year	2nd year	3rd year	Total
**i.**	**Staff (With annual increase of 10%)**	**2340000**	**2574000**	**2831400**	**7745400**
	Scientist-B (Non-Medical)- 1 post @ Rs. 48,000/-	5,76,000	6,33,600	6,96,960	1906560
	Scientist-B (Veterinary)- 1 post @ Rs. 48,000/-	5,76,000	6,33,600	6,96,960	1906560
	Research Assistant – 1 Post @ Rs. 31,000/-	3,72,000	4,09,200	4,50,120	1231320
	Field Assistant - 2 posts @ Rs. 17,000/-	4,08,000	4,48,800	4,93,680	1350480
	Laboratory Assistant -1 post @ Rs. 17000/-	2,04,000	2,24,400	2,46,840	675240
	Data Entry Operator (A) - 1 Posts @ Rs. 17,000/-	2,04,000	2,24,400	2,46,840	675240
**ii.**	**Contingencies**				
	**a. Recurring**	**850000**	**825000**	**625000**	**2300000**
	- Development and printing of forms	25000	0	0	25000
	- Stationary	25000	25000	25000	75000
	- Media and reagents	500000	500000	300000	1300000
	- Glassware and plastic ware	300000	300000	300000	900000
	**b. Non-recurring (equipment)** (Laptop – 1 nos., LCD Projector – 1 nos. & 2 PC systems)	**2,00,000**	**0**	**0**	**200000**

		**c. Travel & Accomodation**	**1,00,000**	**2,00,000**	**2,00,000**	**500000**
**iii.**		**Training & Workshops -**	**272000**	**583000**	**368000**	**1223000**
		13 Trainings at district level (Yr-1=0,Yr-2=10,Yr-3=3) ; 12 MDC meetings (Yr-1=4, Yr-2=4, Yr-3=4) ; 38 Team meetings (Yr-1=19, Yr-2=10, Yr-3=9) ; 50 participants on avg. per training				
	-	Venue Hire @ 5000 per training	0	50,000	15,000	65000
	-	Support Staff @ 1000 per training/meeting	4000	14,000	7000	25000
	-	Training Kit @ Rs. 200 / participant	0	100000	30000	130000
	-	Training Logistics @ Rs. 500 per participant	0	2,50,000	75,000	325000
	-	Banner	1000	5000	2000	8000
	-	AV Support (@2000 per meeting/training)	8000	28000	10000	46000
	-	Team Meetings Logistics @ Rs. 500 per participant (8 members in a team on avg)	76000	40000	36000	152000
	-	MDC Meetings Logistics @ Rs.500 per participant	30000	30000	30000	90000
	-	Dissemination Workshop Logistics (@1200 per person including venue x 60 persons)	0	0	100000	100000
	-	Resource Person Fees (For external experts/advisors) @Rs. 3000 per person per day; Total external experts =8; number of days =36; (51 Units in yr-1, 22 in Yr-2, 21 in Yr-3)	153,000	66,000	63,000	282000
**iv.**		**IEC/BCC activities** (Printing of materials, dissemination and mass media campaigns)	**1,00,000**	**3,00,000**	**3,00,000**	**700000**
**v.**		**Overhead Charges**	0	0	0	**0**
		Total	3862000	4482000	432440	**12668400**

11. Budget justification

(I) Staff

i. Scientist-B - The scientist B is needed to lead the team on site for the duration of the study and provide technical expertise and feedback for project activities. One scientist from public health background and one from veterinary background will be preferred.

ii. Project Assistant: The project assistant shall be responsible for co-ordination of all the project activities, diagnostics, trainings, meetings, logistics and handling of other incidentals and equipment as and where required by the project team.

iii. Field assistants- The 2 field assistants shall be involved in data collection and surveillance system operationalization at the block levels. There are 14 blocks in the study district there by the requirement for 2 FAs with 7 blocks each.

iv. Lab assistant- The person shall be responsible for co ordination of sample collection and analysis of the samples at the referral lab.

v. Data Entry Operator: The DEO shall be responsible for handling data supplied by the selected teams of short term projects and shall compile the same in the format designed for analysis. He/she shall also be managing the database of alumni and shall follow up the participants as per requirement.

(II) *Contingencies:*

i. Stationary- Office stationary shall be required for general use of the staff for the duration of the project. These would include paper, printing cartridges, registers, files etc. for printing of forms and questionnaires.

ii. Recurring consumables like media, glassware and other minor equipment is needed for the diagnostic facility at the state level and collection of samples from the field.

(III) *Travel and accommodation:* Travel to the project site and travel within the district for data and sample collection has been budgeted along with travel costs for the external RPs.

(IV) *Equipments:* A laptop PC and an LCD projector along with screen are required for the conduct of the trainings. A system for data entry operator and a system for the computer programmer are also needed.

(V) *Training and workshops*- Capacity building trainings would be an integral component of our intervention. 13 Trainings at district level (Yr-1=0,Yr-2=10,Yr-3=3) ; 12 MDC meetings (Yr-1=4,

Yr-2=4, Yr-3=4) and 38 Team meetings (Yr-1=19, Yr-2=10, Yr-3=9) have been planned with an average participation of 50 participants per training. Trainings kits will be provided to the participants which would include printed and bound training manual, folders, pens, notepads and other colour printed aids as developed by the TOT for the purpose of the training. The conduct of training shall require other consumables and logistics like arrangement for refreshments, banner etc. We shall be engaging multiple expert external resource persons for activities under various teams.
