# Supplementary material for: One Health approach for elimination of human anthrax in a tribal district of Odisha: Study protocol
Source: PLoS One. 2021 May 27;16(5):e0251041. doi: 10.1371/journal.pone.0251041 (PMC8158997; doi:10.1371/journal.pone.0251041)
Supplement: S4 Appendix — (PDF) [file pone.0251041.s004.pdf]

Directorate of Health Services, Odisha

E mail id :- shrmubbsori@gmail.com , Ph No.-0674-2396827

Letter No. 155 **/PMU /187/17** **Date** 27.08.19

From

Dr. Bishnu Prasad Mohapatra,
Addl. DHS (HRH&R) & Member Convener,
Research & Ethics Committee

To

The Director, Health Services, Odisha
The Director, Medical Education & Training, Odisha
The Director, Public Health, Odisha
The Director, Family Welfare, Odisha
The Director, Nursing, Odisha
The Director, Blood Safety, Odisha
The Director, Food Safety, Odisha
The Director, Vital Statistics & Health Intelligence, Odisha
The Director, SIH&FW, Odisha
The Director, RMRC, BBSR
The Law Officer, Health & FW Deptt., Odisha
The Prof. Community Medicine, SCB MCH, Cuttack

Sub: Minutes of the Research & Ethics Committee Meeting held on 14.08.19

Madam/ Sir,

I am directed to communicate herewith the minutes of the Research & Ethics Committee Meeting held on 14.08.19 at 12.30 P.M. in the Conference Hall of H & FW Deptt., GoO for favour of your information and necessary action.

Yours faithfully,

Mohapatra 27/8/19

Addl. DHS (HRH&R) & Member Convener
Research & Ethics Committee

Memo No..........156......**/ PMU** **Dt.** 27/08 **/2019**

Dr.(Mrs.)Ashoka Mohapatra, AIIMS, Bhubaneswar/ Dr.Bhagirathi Dwibedi, AIIMS, Bhubaneswar/Dr.Leena Das, SCB Medical College & Hospital, Cuttack/Dr. Sanghamitra Pati, RMRC, Bhubaneswar/Dr.Kavi Nila David Retina, AIIMS, Bhubaneswar/ Prof.(Dr.) Santanu Kumar Tripathy, Central Ayurveda Research Institute for Hepatobiliary Disorders, Bhubaneswar for information and necessary action.

Mohapatra 27/8/19

Addl. DHS (HRH&R) & Member Convener
Research & Ethics Committee

Memo No..........157......**/ PMU** **Dt.** 27/08 **/2019**

Copy forwarded to the Directors of Capital Hospital, Bhubaneswar and RGH, Rourkela /All Chief District Medical & Public Health Officers for information & necessary cooperation for data collection in connection with the above research projects.

Mohapatra 27/8/19

Addl. DHS (HRH&R) & Member Convener
Research & Ethics Committee

Memo No.........158........./ PMU Dt. 27/08 /2019

Copy forwarded to Nodal Officer, Cancer Care/ Additional Director (PH)/ State Extended Programme of Immunisation Officer for information.

Addl. DHS (HRH&R) & Member Convener
Research & Ethics Committee

Memo No.........159........./ PMU Dt. 27/08 /2019

Copy forwarded to the Special Secretary (MS)/ Special Secretary (PH) to Govt., H&FW Deptt. for favour of information.

Addl. DHS (HRH&R) & Member Convener
Research & Ethics Committee

Memo No.........160........./ PMU Dt. 27/08 /2019

Copy forwarded to the Mission Director, NHM, Odisha for favour of information.

Addl. DHS (HRH&R) & Member Convener
Research & Ethics Committee

Memo No.........161........./ PMU Dt. 27/08 /2019

Copy forwarded to PS to the Commissioner-cum- Secretary, Health & Family Welfare Department, Govt. of Odisha for kind appraisal of the Commissioner-cum- Secretary, the Chairperson of Research & Ethics Committee.

Addl. DHS (HRH&R) & Member Convener
Research & Ethics Committee

Minutes of the Research & Ethics Committee Meeting held on 14.08.2019

The Research & Ethics Committee Meeting was held on 14.08.2019 at 12.30 P.M. in the conference hall of Health & Family Welfare Deptt., Odisha under the Chairpersonship of Commissioner-cum-Secretary, Health & Family Welfare Department, Govt. of Odisha.

The list of members who attended the meeting is attached at Annexure-I.

At the outset of the meeting, Additional Director (HRH & Research)-cum-Member Convener of Research & Ethics Committee welcomed all the members and briefed the house on different proposals received. The representatives of different institutions who submitted the Research proposals shared their topics through power point presentations followed by open house discussion. The following decisions were taken.

Sl. No.	Research Proposals/study reports	Decision taken
1.	Rota viral diarrhoea among children aged six months to five years- Post Rota vaccination scenario in Khurda district of Odisha- Dr.(Mrs.) Ashoka Mohapatra, AIIMS, Bhubaneswar	• Approved, but subject to approval of Internal Ethics Committee of AIIMS, Bhubaneswar. • Study report should be shared with Health & Family Welfare Deptt., Govt. of Odisha before publication in the public domain.
2.	Protective antibody response following double dose measles vaccination and factors for non-response among tribal children in remote areas of Odisha - Dr.Bhagirathi Dwibedi, AIIMS, Bhubaneswar	Approved • Study report should be shared with Health & Family Welfare Deptt., Govt. of Odisha before publication in the public domain.
3.	LIFE: Low-birth weight Infant Feeding Exploration - Dr.Leena Das, SCB Medical College & Hospital, Cuttack	Approved • Study report should be shared with Health & Family Welfare Deptt., Govt. of Odisha before publication in the public domain.
4.	'One Health' strategy for elimination of human anthrax from an endemic district of Odisha: a demonstration project - Dr.Sanghamitra Pati, RMRC, Bhubaneswar	Approved • Study report should be shared with Health & Family Welfare Deptt., Govt. of Odisha before publication in the public domain.
5.	National Environmental Health Profile: 20 city multisite study- Dr.Sanghamitra Pati, RMRC, Bhubaneswar	Approved • Study report should be shared with Health & Family Welfare Deptt., Govt. of Odisha before publication in the public domain.

6.	Palliative care needs and its existing services in Khurda district - A cross sectional study - Dr.Kavi Nila David Retina, AIIMS, Bhubaneswar	Approved<br>• The title of the study may be revisited as per the suggestion of the committee.
7.	Clinical Evaluation of AYUSH-SL in chronic Filarial Lympodema in patients receiving Mass Drug Administration (MDA) - Prof.(Dr.) Santanu Kumar Tripathy, Central Ayurveda Research Institute for Hepatobiliary Disorders, Bhubaneswar	Approved<br>• Study shall be carried out only in Ayurvedic Dispensary and Ayurvedic Medical College & Hospital under the supervision of an Ayurvedic Medical Officer.<br>• Community and Co-located OPDs in CHCs & PHCs shall not be utilised for the study purpose.<br>• Study report should be shared with Health & Family Welfare Deptt., Govt. of Odisha before publication in the public domain.

The meeting ended with vote of thanks to the chair and participants.

Commissioner-cum-Secretary
Chairperson, Research & Ethics Committee,
H&FW Deptt.Govt. of Odisha

Annexure-I

1. Dr.P.K.Meherda, IAS, Commissioner-cum-Secretary, H&FW Deptt.
2. Dr.B.K.Brahma, Spl. Secretary (MS) to Govt., H&FW Deptt.
3. Dr.H.P.Pattanaik, Spl. Secretary (PH) to Govt., H&FW Deptt.
4. Dr.D.K.Sarangi, Director, Health Services, Odisha
5. Dr.Sushil Kumar Kar, Director , Nursing, Odisha
6. Dr.S.P.Meher, Director, HI&VS, Odisha
7. Dr.S.K.Nayak, Director, Family Welfare, Odisha
8. Dr.Barish Kumar Das, Director, SIHFW, Odisha
9. Dr.M.R.Samantaray, Director, Blood Safety, Odisha
10. Dr.Ajit Kumar Mohanty, Director, Public Health, Odisha
11. Dr.Sanghamitra Pati, Director, RMRC , Bhubaneswar
12. Dr.Prameela Baral, AD, MH, Odisha, Odisha
13. Dr.B.P. Mohapatra, AD (HRH & Research)
14. Dr.Tapas Kumar Patra, SEPIO, Odisha
15. Dr.Dinabandhu Sahoo, Team Leader, SHSRC, NHM
16. Dr.Manoranjan Jena, SCBMCH, Cuttack

Representatives of the Organizations who have submitted research proposals

1. Dr. Sanghamitra Panda, City Hospital, Cuttack
2. Dr. Veena Herekar, JNMC Research Unit, Belagaon
3. Dr.Bhagirathi Dwibedi, AIIMS, Bhubaneswar
4. Dr. Kavi Nila David Retina, AIIMS, Bhubaneswar
5. Dr. Ashoka Mohapatra, AIIMS, Bhubaneswar
6. Ms. Rosalini Tarai, AIIMS, Bhubaneswar
7. Dr. Debdutta Bhattacharya, RMRC, Bhubaneswar
8. Dr. Jaya Singh Kshatri, RMRC, Bhubaneswar
9. Dr. Gurucharan Bhuyan, CARIHD, Bhubaneswar
10. Dr. Kshirod Kumar Ratha, CARIHD, Bhubaneswar
11. Dr. Jayaram Hazra, CARIHD, Bhubaneswar
