# Supplementary material for: One Health approach for elimination of human anthrax in a tribal district of Odisha: Study protocol
Source: PLoS One. 2021 May 27;16(5):e0251041. doi: 10.1371/journal.pone.0251041 (PMC8158997; doi:10.1371/journal.pone.0251041)
Supplement: S5 Appendix — (PDF) [file pone.0251041.s005.pdf]

REGIONAL MEDICAL RESEARCH CENTRE (ICMR), BHUBANESWAR
INSTITUTIUONAL HUMAN ETHICS COMMITTEE
IRB No: ECR/911/Inst/OR/2017

Protection of Human Subjects
IEC Certification/ Declaration of Exemption

1. Request Type
   - ☐ Original
   - ☐ Continuation
   - ☐ Exemption

2. Type of Programme
   - ☐ Research ☐ Contract Research ☐ Collaborative
   - ☐ Institutional Activity - Out Patient Care/ Surveillance/ Referral & Outbreak Investigation ☐ Fellowship ☐ Others

2. Proposal Identification No.
   - Institutional ID :
   - Funding Agency & ID (If any) :

3. Title of Research or Activity: **"One Health" strategy for elimination of human anthrax from an endemic district of Odisha: a demonstration project**

4. Principal Investigator : **Dr. Sanghamitra Pati, Director**

5. Key Personnel at RMRC Bhubaneswar & Role: **Dr S Pati, Director.**

6. Certification of IRB review
   This activity/ proposal has been reviewed and approved by the IEC in accordance with the Indian Council for Medical Research (ICMR), guidelines and other GCP recommendations, by full IRB review or expedited review on **02/03/2019** [DD/MM/YYYY].

7. Remarks (If any) :
   - Confidentially to be maintained
   - Consent to be obtained

8. This activity/ proposal has been scrutinized and declared for exemption for review by IEC as there is less than minimal risk and no linked identifiers.

Dr. Prakash Kumar Sahoo
Scientist- C, Member Secretary
Name & Designation of the Official

Signature:

Date: **02/03/2019**
