# Supplementary material for: One Health approach for elimination of human anthrax in a tribal district of Odisha: Study protocol
Source: PLoS One. 2021 May 27;16(5):e0251041. doi: 10.1371/journal.pone.0251041 (PMC8158997; doi:10.1371/journal.pone.0251041)
Supplement: S6 Appendix — (PDF) [file pone.0251041.s006.pdf]

International Clinical Trials Registry Platform
Search Portal

Main

Note: This record shows only 22 elements of the WHO Trial Registration Data Set. To view changes that have been made to the source record, or for additional information about this trial, click on the URL below to go to the source record in the primary register.

**Register:**	CTRI
**Last refreshed on:**	17 August 2020
**Main ID:**	CTRI/2020/05/025325
**Date of registration:**	22-05-2020
**Prospective Registration:**	Yes
**Primary sponsor:**	Indian Council of Medical Research
**Public title:**	One health approach for elimination of human anthrax in a tribal district of Odisha
**Scientific title:**	One health strategy for elimination of human anthrax from an endemic district of Odisha: a demonstration project
**Date of first enrolment:**	15-06-2020
**Target sample size:**	4060
**Recruitment status:**	Not Yet Recruiting
**URL:**	http://www.ctri.nic.in/Clinicaltrials/pmaindet2.php?trialid=43789
**Study type:**	Interventional
**Study design:**	Cluster Randomized Trial Method of generating randomization sequence:Computer generated randomization Method of allocation concealment:Not Applicable Blinding and masking:Not Applicable
**Phase:**	N/A

Countries of recruitment

India

Contacts

Name: Dr Dedutta Bhattacharya
Address: ICMR RMRC Chandrashekarpur Bhubaneswar 751023 Khordha, ORISSA India
Telephone:
Email: drdebdutta.bhattacharya@yahoo.co.in
Affiliation: ICMR Regional Medical Research Centre

Name: Dr Dedutta Bhattacharya
Address: ICMR RMRC Chandrashekarpur Bhubaneswar 751023 Khordha, ORISSA India
Telephone:
Email: drdebdutta.bhattacharya@yahoo.co.in
Affiliation: ICMR Regional Medical Research Centre

Key inclusion & exclusion criteria

Inclusion criteria: Only adults
Exclusion criteria: Below to age of 18 years

Age minimum:
Age maximum:
Gender:

Health Condition(s) or Problem(s) studied

Intervention(s)

Intervention1: One Health Strategy for Elimination of human anthrax: Strengthening the health care and surveillance

system for early reporting of suspected cases of human and animal anthrax by developing and implementing the â?? One-Healthâ?? strategy. Establishment of an anthrax diagnosis facility in the state to facilitate early diagnosis and reporting of anthrax cases based on standardized case definitions. Provision of post-exposure prophylaxis of suspected contacts by the health department at the health facility and community. Placing a framework for categorization of geographical â??Risk-Zonesâ?? based on GIS mapping of cases for prioritization of public health interventions. Development of departmental and interdepartmental Standard Operating Procedures for both exclusive and inter-operable activities and conduct of multi departmental coordination meetings as per mutually agreed calendar. Capacity building of stakeholders from various departments will be done through orientation, training and sensitization workshops. Development of capacity building material for each stakeholder group, finalization of activity calendar and micro plan followed by conduct orientation & training at district level and sensitization workshops at sector/gram panchayat/PHC level for capacity building among stakeholders from various departments of the district such as Health, Veterinary and Forest.
Control Intervention1: Not Applicable: Not Applicable

Primary Outcome(s)

Primary outcome to see the effectiveness of public health interventions for elimination of Human anthrax cases in the District.Timepoint: Baseline survey in third and fourth quarter of the study period. End line survey in the ninth and tenth quarter.

Secondary Outcome(s)

1. To see effectiveness of coordinated activities of the different departments (Health, Veterinary & Forest), 2. Impact of Capacity building of stakeholders, 3. Early diagnosis of cases and surveillance mechanism. 4. Improvement in knowledge, attitude and practice of community people. 5. To assess the behavioral changes in the food habits of community people etc.Timepoint: At the end of 2nd year and 3rd year of the study period.

Secondary ID(s)

NIL

Source(s) of Monetary Support

ICMR RMRC Bhubaneswar

Secondary Sponsor(s)

Ethics review

Status: Approved
Approval date: 02/03/2019
Contact:
Institutional Human Ethics Committee ICMR RMRC Bhubaneswar

Status: Approved
Approval date: 27/08/2019
Contact:
Research and Ethics Committee of Directorate of Health Services Odisha

Results

Results available:
Date Posted:
Date Completed:
URL:

Disclaimer: Trials posted on this search portal are not endorsed by WHO, but are provided as a service to our users. In no event shall the World Health Organization be liable for any damages arising from the use of the information linked to in this section. None of the information obtained through use of the search portal should in any way be used in clinical care without consulting a physician or licensed health professional. WHO is not responsible for the accuracy, completeness and/or use made of the content displayed for any trial record.

Copyright - World Health Organization - Version 3.6 - Version history
