# Supplementary material for: One Health approach for elimination of human anthrax in a tribal district of Odisha: Study protocol
Source: PLoS One. 2021 May 27;16(5):e0251041. doi: 10.1371/journal.pone.0251041 (PMC8158997; doi:10.1371/journal.pone.0251041)
Supplement: S7 Appendix — (DOC) [file pone.0251041.s007.doc]

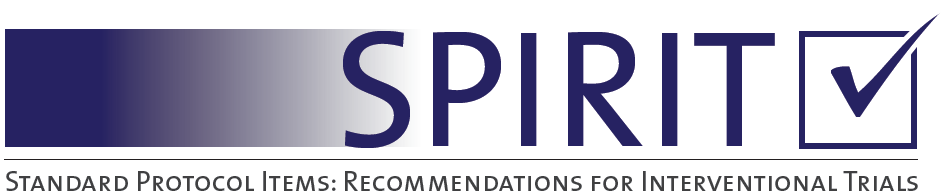


SPIRIT 2013 Checklist: Recommended items to address in a clinical trial protocol and related documents*

| Section/item | ItemNo | Description |
| --- | --- | --- |
| **Administrative information** | | |
| Title | 1 | One Health approach for elimination of human anthrax in a tribal district of Odisha: A demonstration project |
| Trial registration | 2a | This trial was prospectively registered with the Clinical Trials Registry of India **[**CTRI/2020/05/025325] on 22 May 2020. |
|  | 2b | N/A |
| Protocol version | 3 | N/A |
| Funding | 4 | External government funding body from India for all pre budgeted financial support (Indian Council of Medical Research, New Delhi) |
| Roles and responsibilities | 5a | ICMR – Regional Medical Research Centre, Bhubaneswar, Odisha is responsible for overall implementation of the intervention trial. Department of Community Medicine, Saheed Laxman Nayak Medical College & Hospital Koraput is responsible for support in preparation and execution of capacity building trainings for stakeholders from health, veterinary and forest departments in Koraput. |
|  | 5b | Indian Council of Medical Research, Ansari Nagar New Delhi 110029 |
|  | 5c | Funding body was not involved in the design of the study and collection, analysis, and interpretation of data, or in writing the manuscript. |
|  | 5d | N/A |
| Introduction |  |  |
| Background and rationale | 6a | Background, Paragraph 2 |
|  | 6b | N/A |
| Objectives | 7 | Background, Objectives |
| Trial design | 8 | Methods, Paragraph 1 |
| Methods: Participants, interventions, and outcomes | | |
| Study setting | 9 | Methods, Setting |
| Eligibility criteria | 10 | Abstract, Methods |
| Interventions | 11a | Methods, Overview of intervention package |
|  | 11b | N/A |
|  | 11c | N/A |
|  | 11d | N/A |
| Outcomes | 12 | Primary, secondary, and other outcomes, including the specific measurement variable (eg, systolic blood pressure), analysis metric (eg, change from baseline, final value, time to event), method of aggregation (eg, median, proportion), and time point for each outcome. Explanation of the clinical relevance of chosen efficacy and harm outcomes is strongly recommended |
| Participant timeline | 13 | Methods, Study timeline (see Table - 2) |
| Sample size | 14 | The sample size required for the quantitative component of baseline (and end line) study was estimated to be 2640. This was calculated by the following formula:    Minimum Sample Size Required = [(1.96)^2^*P*(1-P)]  ---------------------------------- * (Design Effect)  d^2^  Where,  Level of confidence assumed at 95%, corresponding to Z=1.96;  P= Prevalence of exposure to anthrax (Assumed at 5%)  d= Relative precision at 20% of P  Design effect = 1.3.  Non Response rate = 10% |
| Recruitment | 15 | Computer randomised formula and planning to completed the recruitment within the given timeline. Recruitment will take place in coordination with health department in the district for support in identification and will be done by our trained field investigators. |
| **Methods: Assignment of interventions (for controlled trials)** | | |
| Allocation: |  |  |
| Sequence generation | 16a | Through computer-generated random numbers |
| Allocation concealment mechanism | 16b | N/A |
| Implementation | 16c | N/A |
| Blinding (masking) | 17a | N/A |
|  | 17b | N/A |
| **Methods: Data collection, management, and analysis** | | |
| Data collection methods | 18a | Assessment and collection of outcome will be done through baseline, and end line surveys. |
|  | 18b | Random participants will be covered from the communities in baseline and end line. |
| Data management | 19 | Data is being collected through ODK App (GIS Collect) and procedures can be found in the Methods, Baseline paragraph in manuscript. |
| Statistical methods | 20a | Methods, Data Analysis paragraph |
|  | 20b | N/A |
|  | 20c | N/A |
| **Methods: Monitoring** | | |
| Data monitoring | 21a | We have formed a separate team for Monitoring and evaluation of data and study procedures. Supervisor at field level will make sure the quality of data collection in the field on daily basis. |
|  | 21b | The interim analysis will be done for quality checks in between the baseline and end line. |
| Harms | 22 | N/A |
| Auditing | 23 | N/A |
| Ethics and dissemination | | |
| Research ethics approval | 24 | Approved by the Institutional and state ethics committees |
| Protocol amendments | 25 | Any changes in the protocol will be presented to concerned ethics committee for their approval before taking it into the action. |
| Consent or assent | 26a | All the participants will complete the written consent form process before participation in study and also they will receive a written information about the study for their reference. Mentioned in the Declaration in manuscript. |
|  | 26b | N/A |
| Confidentiality | 27 | All the collected personal information of the participants will be kept confidential and data will not be presented anywhere with their personal identity. The information will be with primary institution only and will not be shared with anybody. |
| Declaration of interests | 28 | The authors declare that they have no competing interests |
| Access to data | 29 | Access of data will be with study Investigators only. |
| Ancillary and post-trial care | 30 | N/A |
| Dissemination policy | 31a | If trial got successful than it will suggested to the state and central government in India for replication of the protocol to eliminate same cause in rest of the parts of the state and country. Also dissemination will be done via publication, reporting in results databases to the funders. |
|  | 31b | Only to the Investigators and project staffs. |
|  | 31c | All the outcomes will be made publically available after publication with open access. |
| Appendices |  |  |
| Informed consent materials | 32 | Consent form developed by the investigators and approved by the Institutional ethics committee will be taken from all participants. |
| Biological specimens | 33 | Swab samples of anthrax infected people in the district will be collected by following the SOP developed by the investigators. |

*It is strongly recommended that this checklist be read in conjunction with the SPIRIT 2013 Explanation & Elaboration for important clarification on the items. Amendments to the protocol should be tracked and dated. The SPIRIT checklist is copyrighted by the SPIRIT Group under the Creative Commons “[Attribution-NonCommercial-NoDerivs 3.0 Unported](http://www.creativecommons.org/licenses/by-nc-nd/3.0/)” license.
