# Supplementary material for: One Health approach for elimination of human anthrax in a tribal district of Odisha: Study protocol
Source: PLoS One. 2021 May 27;16(5):e0251041. doi: 10.1371/journal.pone.0251041 (PMC8158997; doi:10.1371/journal.pone.0251041)
Supplement: S8 Appendix — (PDF) [file pone.0251041.s008.pdf]

Confidential – For Research Propose Only

ICMR – REGIONAL MEDICAL RESEARCH CENTRE BHUBANESWAR

One Health strategy for elimination of human anthrax from endemic district of Odisha: a demonstration project

BASELINE QUESTIONNAIRE

**Socio-Demographic Characteristics**			
Q1	Name of Interviewer-		
Q2	Name of Interviewee-		
Q3	Date/Time:		
Q4	Age:		
Q5	Gender:	1. Male<br>2. Female<br>3. Others	
Q6	Block:		
Q7	Town /Village:		
Q8	GIS:		
Q9	Marital status:	1. Single<br>2. Married<br>3. Divorced<br>4. Widowed<br>5. Separated	
Q10	No. of members in family:	---------	
Q11	Type of family:	1. Joint<br>2. Nuclear	
Q12	Religion:<br>*INTERVIEWER: allow the respondent to reply without reading categories. Clarify as needed.*<br>*Only one option allowed*	1. Hindu;<br>2. Muslim;<br>3. Christian;<br>4. Sikh;<br>5. Others(specify)	
Q13	Caste:	1. SC<br>2. ST<br>3. OBC<br>4. General	
Q14	Educational qualification:	Years of schooling---------	
Q15	Occupation:	1. Unemployed<br>2. Government Service<br>3. Private Service<br>4. Housewife<br>5. Agriculture<br>6. Business<br>7. Daily labor<br>8. Others (Specify)	
Q16	What is the annual income of family?	Income in Rs.----------------	

**Information on domestic animal**			
Q17	Do you keep livestock?	1. Yes<br>2. No	***(If NO, then skip to Q48)***

Q18	Who deals with the livestock?	1. Myself<br>2. Wife/husband<br>3. Son<br>4. Daughter<br>5. Son-in-law<br>6. Daughter-in-law<br>7. Grandchild<br>8. Parent<br>9. Parent-in-law<br>10. Brother<br>11. Sister<br>12. Brother-in-law<br>13. Sister-in-law<br>14. Other relative	
Q19	Which livestock do you have?	1. Cow/Buffalo<br>2. Goat<br>3. Pig<br>4. Sheep<br>5. Other(specify)	
Q20	What is the purpose for keeping livestock?	1. Leather industry<br>2. Wool<br>3. Skinning<br>4. Dairy<br>5. Farming<br>6. Selling meat<br>7. Others (Specify)	
Q21	Where do you keep your livestock at your residence?	1. Separate animal shed<br>2. Inside the house<br>3. Other (specify)	
Q22	Where do you take the livestock for grazing?	1. Forest<br>2. Agricultural land<br>3. Grass field<br>4. Buy Commercial fodder<br>5. Others(specify)	
Q23	How many times do you take them for grazing in a day?	1. Once<br>2. Twice<br>3. Thrice<br>4. Other (Specify)	
Q24	How do you dispose the solid & animal waste products?	1. Dispose in water bodies<br>2. Residing place<br>3. Dig a pit<br>4. Throw anywhere<br>5. Common pit<br>6. Others(specify)	
Q25	How many years of experience do you have of handling livestock animals?	1. Less than one year<br>2. 1-5 years<br>3. 5-10 years<br>4. More than 10 years	

**Vaccination**			
Q26	Are you aware of vaccination of livestock?	1. Yes<br>2. No	***(If No, then skip to Q38)***
Q27	Is the vaccination done as per schedule or as per disease?	1. As per schedule<br>2. As per disease<br>3. Both	**If 2 then skip to Q38**

Q28	Have you ever vaccinated your livestock animals?	1. Yes<br>2. No	***(If No, then skip to Q38)***
Q29	Which animals are vaccinated?	1. Cow/Buffalo<br>2. Goat<br>3. Pig<br>4. Sheep<br>5. Other(specify)	
Q30	Do you want your animals to be vaccinated?	1. Yes<br>2. No	***(If No, then skip to Q32)***
Q31	If **Yes**, then why you want the animals to be vaccinated?	1. To keep animals healthy/disease free<br>2. For longevity of animals<br>3. For better off springs<br>4. Others (Specify)	
Q32	If **No**, then why don't you want the animals to be vaccinated?	1. Productivity of animals will be hampered<br>2. Vaccination is costly<br>3. Veterinary doctor/ LI inspector is not coming home<br>4. Others (Specify)	
Q33	If as per schedule, when did you take the animals for vaccination?	1. Less than 6 months<br>2. 6 months - 1 year<br>3. 1-2 years<br>4. More than 2 years	
Q34	Where do you take the animals for vaccination?	1. Veterinary hospital<br>2. Home<br>3. Animal health checkup camps<br>4. Others (Specify)	
Q35	Who does the vaccination of animals?	1. Veterinary doctor<br>2. Livestock Inspector<br>3. Other (Specify)	
Q36	Is the vaccination free of cost?	1. Yes<br>2. No	***(If Yes, then skip to Q38)***
Q37	If **No**, then how much do you spend on vaccination per animal?	Specify--------------(in Rs.)	

**Animal Handling**			
Q38	Any recent deaths of animals in last 2 years?	1. Yes<br>2. No	***(If No, then skip to Q41)***
Q39	If yes, then when did it occur?	1. 0 - 6 months<br>2. 6 months – 1 year<br>3. 1 year – 1.5 years<br>4. 1.5 – 2 years	
Q40	What were the symptoms during death?	1. High fever<br>2. Blood from nose, mouth and anus<br>3. Breathing problems<br>4. Tremor before death<br>5. No symptoms<br>6. Don't know	
Q41	Do you skin dead animals?	1. Yes<br>2. No	***(If No, then skip to Q44)***
Q42	If yes do you wear protective equipments for it?	1. Yes<br>2. No	
Q43	What protective equipments do you wear?	1. Face mask<br>2. Gloves<br>3. Head cover<br>4. Body wear	

		5. Others (Specify)	
Q44	What do you do with the dead bodies of animal/ livestock?	1. Burial<br>2. Throw them away<br>3. Distribute among villagers<br>4. Selling the carcass<br>5. Report to closest veterinary office<br>6. Incineration<br>7. Consume the meat<br>8. Others(specify)	
Q45	Do you know about getting financial help from the Govt. for burial of animals?<br><br>*If yes, then move to next questions.*	1. Yes<br>2. No<br>3. Don’t know	***(If No or Don’t’ Know, then move to Q48)***
Q46	Do you think compensation amount is enough for reporting the suspected dead animal?	1. Yes<br>2. No<br>3. Don’t know	
Q47	Have you ever received compensation for reporting anthrax cases to the relevant authority?	1. Yes<br>2. No<br>3. Don’t know	

**Food Habits**			
Q48	Do you consume meat?	1. Yes<br>2. No	***(If No, then skip to Q57)***
Q49	What type of meat do you consume?	1. Beef<br>2. Pig<br>3. Goat<br>4. Sheep<br>5. Others (Specify)	
Q50	What is the source of meat?	1. Market<br>2. Hunting<br>3. Livestock animals<br>4. Dead animals<br>5. Others (Specify)	
Q51	How do you consume the meat?	1. Cooked<br>2. Roasted<br>3. Raw<br>4. Dried<br>5. Others(specify)	
Q52	How often do you consume the meat?	1. Once in a week<br>2. Twice in week<br>3. Occasionally<br>4. Daily	
Q53	Do you preserve your meat?	1. Yes<br>2. No	***(If No, then skip to Q55)***
Q54	If yes, how do you preserve your meat? (Explain by respondent)	1. Refrigerate<br>2. Keep in water<br>3. Dried<br>4. Any traditional methods	
Q55	Do you take animal blood?	1. Yes<br>2. No	***(If No, then skip to Q57)***
Q56	If yes, how do you consume?	1. Cooked<br>2. Dried<br>3. Raw<br>4. Others(specify)	

**Knowledge assessment & awareness of the respondent about anthrax**			
Q57	Have you ever heard about anthrax?	1. Yes<br>2. No	***(If No, then go to the end)***
Q58	If yes, from where did you come to know about anthrax?	1. Newspaper<br>2. Doctor<br>3. ANM/AWW/ASHA<br>4. Community<br>5. Forest Department<br>6. Veterinary Department<br>7. Others(specify)	
Q59	Do you have any local names for anthrax?	**VERTBATIUM ()**	
Q60	Who all are affected by anthrax?	1. Human<br>2. Livestock<br>3. Both<br>4. None<br>5. Don't Know	
Q61	Do you know how anthrax is transmitted?	1. Yes<br>2. No<br>3. Don't know	***(If No or Don't Know, then skip to Q63)***
Q62	If yes, how it is transmitted?	1. Air<br>2. Food<br>3. Contact with infected animal<br>4. Soil<br>5. Flies and Insects<br>6. All of above<br>7. Don't know<br>8. Others	
Q63	What are the symptoms of anthrax in animals?	1. High fever<br>2. Blood from nose, mouth and anus<br>3. Breathing problems<br>4. Tremor before death<br>5. Don't know<br>6. Others (Specify)	
Q64	How do you respond to anthrax suspected cases in animals?	1. None<br>2. Inform relevant authority<br>3. Disinfect the infected areas<br>4. Treat with antibiotic<br>5. Traditional methods<br>6. Wait and see<br>7. Others (Specify)	
Q65	If you inform, then whom do you do?	1. ASHA/ANM<br>2. Livestock officials<br>3. Forest Department Officials<br>4. PRI members<br>5. Teachers/Headmaster<br>6. Unskilled health workers	
Q66	How do you handle animals dead due to anthrax?	1. Throw them away<br>2. Consume the meat<br>3. Burying<br>4. Burning<br>5. Both burning and burying<br>6. Inform health officials<br>7. Inform veterinary officials<br>8. Others (Specify)	**If 1 then skip to Q67,<br>If 2 then skip to Q68,<br>If 3 then skip to Q69,<br>If 4 then skip to Q70.**

Q67	If you throw the animals, then where do you do?	1. Forest<br>2. Agricultural fields<br>3. Nearby house<br>4. Outside village<br>5. Drainage system<br>6. Water bodies<br>7. Others (Specify)	
Q68	If you consume the meat, then which part of animal body do you consume?	**VERTBATIUM ()**	
Q69	If you bury the animals, then where do you do?	1. Forest<br>2. Agricultural fields<br>3. Nearby house<br>4. Outside village<br>5. Others (Specify)	
Q70	If you burn the animals, then where do you do?	1. Forest<br>2. Agricultural fields<br>3. Nearby house<br>4. Outside village<br>5. Others (Specify)	
Q71	Have you heard about anthrax cases in animals in last two years?	1. Yes<br>2. No	***(If No, then skip to Q74)***
Q72	If yes, then when did it happen?	1. 0 - 6 months<br>2. 6 months – 1 year<br>3. 1 year – 1.5 years<br>4. 1.5 years – 2 years	
Q73	How many cases were there in last two years?	**VERTBATIUM ()**	
Q74	Has any livestock of yours suffered from anthrax?	1. Yes<br>2. No<br>3. Don't Know	
Q75	Is there any activities conducted on anthrax by veterinary department?	1. Yes<br>2. No<br>3. Don't Know	
Q76	If yes, specify.	**VERTBATIUM()**	
Q77	Are you aware about anthrax in humans?	1. Yes<br>2. No	***(If No, then go to the end)***
Q78	How do you think people get infected with anthrax?	1. Contact with infected livestock<br>2. Consumption of meat from infected animal<br>3. Consumption of other products from infected animal<br>4. Contact with wild animals<br>5. Slaughtering an infected carcass<br>6. Don't know<br>7. Others(Specify)	
Q79	What are the symptoms of anthrax in humans?	1. Abdominal pain<br>2. Swollen neck<br>3. Bleeding from all openings<br>4. Ulcers of skin<br>5. Others(specify)<br>6. Don't know	

Q80	Have you heard about anthrax cases in humans in last two years?	1. Yes<br>2. No<br>3. Don’t know	***(If No, then skip to Q83)***
Q81	If yes, then when did it happen?	5. 0 - 6 months<br>6. 6 months – 1 year<br>7. 1 year – 1.5 years<br>8. 1.5 years – 2 years	
Q82	How many cases were there in last one year?	**VERTBATIUM()**	
Q83	Has any member of your family suffered from anthrax?	4. Yes<br>5. No<br>6. Don’t Know	
Q84	Is there any activities conducted on anthrax by health department?	1. Yes<br>2. No<br>3. Don’t Know	
Q85	If yes, specify.	**VERTBATIUM()**	
